# Supplementary figures and images for: Oxygen systems to improve clinical care and outcomes for children and neonates: A stepped-wedge cluster-randomised trial in Nigeria
Source: PLoS Med. 2019 Nov 11;16(11):e1002951. doi: 10.1371/journal.pmed.1002951 (PMC6844455; doi:10.1371/journal.pmed.1002951)

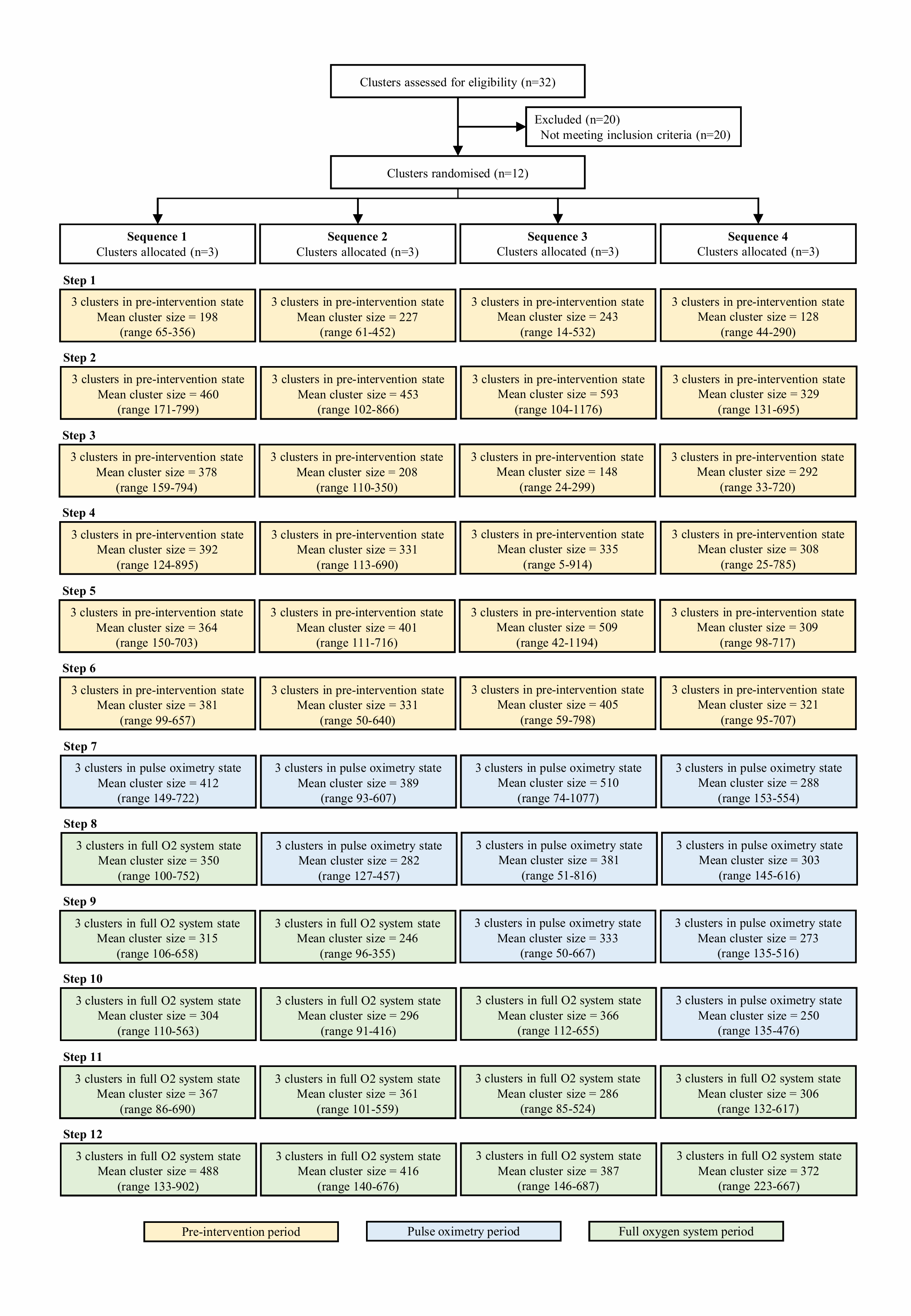

Supplement: S1 Fig — (TIF) [file pmed.1002951.s002.tif]
