# Supplementary material for: Oxygen systems to improve clinical care and outcomes for children and neonates: A stepped-wedge cluster-randomised trial in Nigeria
Source: PLoS Med. 2019 Nov 11;16(11):e1002951. doi: 10.1371/journal.pmed.1002951 (PMC6844455; doi:10.1371/journal.pmed.1002951)
Supplement: S2 Text — (DOCX) [file pmed.1002951.s004.docx]

# **S2 Text – Analysis Plan & Severity-adjusted analysis model**

*Paper: Graham HR, Bakare AA, Ayede AI, et al. Oxygen systems to improve clinical care and outcomes for children and neonates: a stepped-wedge cluster-randomised trial in Nigeria.*

**ANALYSIS PLAN – Effect of the improved oxygen system on clinical and practice outcomes**

**ANALYSIS POPULATIONS**

We will do primary analyses of intervention effect on the following populations: all children (<15 years of age, excluding neonates); all neonates (<28 days of life); children with case definition pneumonia; children with case definition malaria; preterm/small (<37 weeks or <2kg) neonates. We will analyse effectiveness using intention to treat, according to the time hospitals were randomised to cross-over.  We will report any differences between intended crossover time and actual crossover time.

**OUTCOMES**

PRIMARY OUTCOME

The primary outcome is binary – died (yes/no) – where “yes” includes “died in hospital” and “discharged unwell, expected to die”, and “no” includes “discharged well”, “discharged against medical advice, or abscond”, “transferred”.

SECONDARY OUTCOMES

Secondary outcomes include:

- Binary clinical outcomes for “discharged against medical advice, or abscond” and “transferred”.
- Binary practice outcomes for “pulse oximetry performed on admission”, “oxygen administered appropriately on admission”.

**VARIABLES**

INTERVENTION

- Intervention – binary indicator variable for whether the intervention has been introduced into a particular cluster (1=intervention has been introduced, 0=intervention has not been introduced). I.e. this is at the cluster-level (not individual level). Two-week wash-in period at start of month of intervention with admissions during this period dropped from analysis.

CLUSTER

- Cluster – indicator for each Hospital

COVARIATES

- Time (Step) - indicator variables for each step, in 4-month blocks from study commencement.
- Hospital*Time – interaction variable for each Hospital and Time point (Step).

ADDITIONAL COVARIATES (for ‘severity-adjusted’ analysis)

- Marker of severity – use to adjust for individual variation in presentation (e.g. seasonally, diagnostically, between facilities, over time). Assume missing clinical sign is negative. Not hypoxaemia, because confounded with intervention time (pulse oximeters introduced at start of data collection). Select individual clinical signs identified using a logistic regression model to predict death in pre-intervention data (constructed using backward and forward stepwise regression).
- Age (continuous variable in months) – only for Child population (not Newborns)
- Sex (binary variable, male/female)
- Hospital Type (binary variable; 0=government, 1=mission).

**STATISTICAL ANALYSES**

We will report characteristics of the individuals and clusters descriptively, by intervention and control periods, to illustrate potential imbalance between exposed and unexposed and allow for discussion of potential biases. Binary variables will be described using frequencies and proportions; continuous variables will be described using mean/median and ranges. We will illustrate the number of observations within each cluster by overlaying the summary numbers and proportions onto a stepped-wedge diagram.

PRIMARY DATA ANALYSIS

We will use logistic GLMM to do primary and secondary analyses of effect of the intervention. The “basic” GLMM model for primary analysis will analyse individual patient data, with random effects for hospital and hospital*time interaction. The model will have fixed effects for intervention and time (step). We will do sensitivity testing to evaluate whether extensions to the basic Hussey and Hughes models are needed – specifically, using additional random effects for hospital-time interaction {Hemming, 2017 #10487}. We will express the intervention effect as an odds ratio with 95% confidence intervals.

SECONDARY DATA ANALYSES

We will analyse the impact of pulse oximetry by including retrospective data in a similar GLMM model (recognising the limitations due to missing data). This “extended” GLMM model for secondary analysis will also analyse individual patient data, with random effects for hospital and hospital*time interaction. The model will have fixed effects for intervention, time (step), and additional fixed effects for markers of severity, age, sex, hospital type.

We will report severity-adjusted analysis for the primary and secondary analyses (for clinical outcomes, not for practice outcomes).

We will evaluate the primary clinical outcomes on an individual hospital basis using multivariate logistic regression, set up similarly to the GLMM model. We will examine heterogeneity in effects between different hospitals using a random hospital-interaction term and using less formal within-cluster comparisons of exposed and unexposed periods.

SUBGROUP ANALYSES

We will consider subgroup analyses of intervention effect on age subgroups (infant 28 days to 12 months; young child under 5 years; older child 5 to 15 years), other major diagnoses (e.g. neonatal sepsis), and according to disease severity. However, these results will need to be interpreted with caution given the small numbers.

**SEVERITY-ADJUSTED ANALYSIS MODEL**

We did ‘severity-adjusted’ analysis by including signs of illness severity, age, sex, and type of hospital (mission versus government) as additional fixed effects in the analytical model. We did this because we believed the primary clinical outcome (mortality) may be confounded by changes in admission pattern (e.g., hospitals that referred sick patients elsewhere prior to having oxygen available would keep these sicker patients, some of whom would die).

To set up the severity-adjusted model, we first identified candidate variables that may be associated with mortality using univariate logistic regression on pre-intervention data. We then used backward stepwise regression to build a multivariate logistic regression model and isolate which variables might be independent predictors of death. We included variables that were significant to p-value <0.05 as fixed effects in the mixed-effects model that was subsequently used for all clinical outcomes. We did not use the severity-adjusted model for practice outcomes. We conducted this process separately for the neonatal and child data, as there were different candidate variables and we expected differences in which variables would be associated with death.

For neonates we considered the following clinical symptoms and signs (bold items included in the final model):

- Maternal fever during labour (>38 Celsius)
- Prolonged rupture of membranes (>18 hours)
- **Offensive amniotic fluid (meconium stained)**
- **Very poor feeding, or unable to suck**
- **Lethargy, drowsy or unconscious (difficult to wake, moving only when stimulated)**
- **Hypotonia (floppy), or spasms**
- High pitched crying, or inconsolable crying
- Fast breathing (>60 breaths per minue)
- **Severe respiratory distress (e.g. grunting, gasping, severe chest indrawing)**
- **Central cyanosis (blue lips or tongue)**
- Jaundice (yellow eyes or skin)
- **Severe abdominal distension**
- Umbilical (or skin) redness or pus
- Bulging fontanelle

We also included sex, and hospital type (government versus mission hospital) in the mixed-effects model, as we believed these additional covariates may be associated with clinical outcome and intervention effect. (NB: we included sex even though it was not associated with neonatal death in the baseline data because of extensive other data on sex differences in neonatal outcomes^12^). Table 1 shows output from the multiple regression model with the selected clinical signs for neonates.

Table 1 - Estimates from multiple regression model predicting neonatal death using pre-intervention data (<28 days of age)

| **Variable** | **Odds ratio** | **95% confidence interval** |
| --- | --- | --- |
| Offensive amniotic fluid (meconium stained) | 0.41 | 0.24-0.70 |
| Very poor feeding, or unable to suck | 2.69 | 2.25-3.23 |
| Lethargy, drowsy or unconscious (difficult to wake, moving only when stimulated) | 2.46 | 1.77-3.41 |
| Hypotonia (floppy), or spasms | 1.68 | 1.20-2.36 |
| Severe respiratory distress (e.g. grunting, gasping, severe chest indrawing) | 4.35 | 3.53-5.36 |
| Central cyanosis (blue lips or tongue) | 3.20 | 2.30-4.46 |
| Severe abdominal distension | 4.54 | 2.72-7.59 |
| Sex (male compared to female) | 1.04 | 0.88-1.23 |
| Hospital type (mission compared to government) | 2.15 | 1.81-2.55 |
| _cons | 0.03 | 0.03-0.04 |

Notes: Area under ROC curve = 0.7794.

For children we considered the following clinical symptoms and signs (bold items included in the final model):

- **History of fever**
- Cough or difficult breathing
- **Severe respiratory distress (e.g. grunting, gasping, severe chest indrawing)**
- **Central cyanosis (blue lips or tongue)**
- **Pallor (pale conjunctiva or skin)**
- **Jaundice (yellow eyes or skin)**
- Diarrhoea (>3 per day)
- Unable to breastfeed or drink adequately
- **Convulsions (seizures, fits)**
- Confusion, lethargy (difficult to wake)
- **Coma (unconscious or barely conscious)**
- Signs of shock (cold hands, capillary refill ?3 seconds, high heart rate with weak pulse, or low or unmeasurable blood pressure)
- **Signs of severe dehydration (lethargy, sunken eyes, decreased skin turgor)**
- **Severe malnutrition (weight-for-length <-3SD, MUAC <115mm, or oedema of both feet)**

We also included age, sex, and hospital type (government versus mission hospital) in the mixed-effects model, as we believed these additional covariates may be associated with clinical outcome and intervention effect. Table 2 shows output from the multiple regression model with the selected clinical signs for children.

Table 2 - Estimates derived from multiple regression model predicting child death using pre-intervention data (28 days to 14 years of age)

| **Variable** | **Odds ratio** | **95% confidence interval** |
| --- | --- | --- |
| History of fever | 0.66 | 0.53-0.83 |
| Severe respiratory distress (e.g. grunting, gasping, severe chest indrawing) | 4.71 | 3.78-5.86 |
| Central cyanosis (blue lips or tongue) | 9.13 | 3.76-22.16 |
| Pallor (pale conjunctiva or skin) | 1.83 | 1.50-2.23 |
| Jaundice (yellow eyes or skin) | 2.12 | 1.51-3.24 |
| Convulsions (seizures, fits) | 1.52 | 1.19-1.94 |
| Coma (unconscious or barely conscious) | 4.88 | 3.75 |
| Signs of severe dehydration (lethargy, sunken eyes, decreased skin turgor) | 3.34 | 2.38-4.70 |
| Severe malnutrition (weight-for-length <-3SD, MUAC <115mm, or oedema of both feet) | 1.89 | 1.10-3.27 |
| Age (months) | 1.002 | 1.000-1.005 |
| Sex (male compared to female) | 0.77 | 0.64-0.93 |
| Hospital type (mission compared to government) | 1.46 | 1.21-1.77 |
| _cons | 0.02 | 0.02-0.02 |

Notes: Area under ROC curve = 0.7755.
