# Supplementary material for: Oxygen systems to improve clinical care and outcomes for children and neonates: A stepped-wedge cluster-randomised trial in Nigeria
Source: PLoS Med. 2019 Nov 11;16(11):e1002951. doi: 10.1371/journal.pmed.1002951 (PMC6844455; doi:10.1371/journal.pmed.1002951)
Supplement: S1 Table — (DOCX) [file pmed.1002951.s005.docx]

# **S1 Table – Description of the improved oxygen system intervention**

*Graham H, et al. Oxygen systems to improve clinical care and outcomes for children and neonates: a stepped-wedge cluster-randomised trial in Nigeria.*

This table describes the improved oxygen system intervention, as specified in the study protocol, including intervention components, purpose, and description. Pulse oximeters, together with basic oximetry training, were intended to be distributed to all hospitals on commencement of the prospective data collection in November 2015, marking the start of the “pulse oximetry period”. All other aspects of the intervention were implemented at hospitals according to stratified randomisation on pre-specified dates (March 2016, July 2016, November 2016, March 2017), marking the start of the “full oxygen system period”.

| Intervention component | Purpose(s) | Description |
| --- | --- | --- |
| Standardised equipment package  - oxygen concentrator  - pulse oximeter  - oxygen delivery equipment  - maintenance equipment | To enable reliable, continuous access to oxygen for all children and neonates.  To make it easy to use oxygen correctly.  To make it easy to maintain oxygen equipment in good function. | Selection of quality, user-friendly equipment that is proven to function in hot, humid environments and capable of being maintained with minimal technical skill: Airsep Newlife Elite oxygen concentrator, Lifebox pulse oximeter (neonatal and child probes), Airsep Sureflow flowmeter assembly, nasal prongs and tubing, oxygen analyser, installation and maintenance gear. |
| Clinical education and support  - basic oximetry training  - healthcare worker training on the clinical use of oxygen  - supportive supervision (focussed on pulse oximetry and oxygen) | To build healthcare worker capacity and motivation to use oxygen well.  To stimulate healthcare workers to make their work environment more conducive to good clinical care. | Clinical training material based on WHO guidelines[1, 2] and will include: clinical approaches to sick children; recognition and treatment of hypoxia; use of pulse oximeters, oxygen therapy, and oxygen concentrators. Using Merrill’s approach to active learning[3], training will be active, task-based, and intentionally target motivation. Training conducted on-site at each hospital.   - One-hour basic pulse oximetry training for nurses and doctors when pulse oximeters are distributed. - Half-day training on the clinical use of oxygen at the time of installation, using an ‘apprentice’ model where individual leaders are trained, and then they are supervised to train their colleagues (then coordinate re-training). |
| Technical training and support  - technician training on maintenance and repair  - supportive supervision | To build technician capacity and motivation to maintain and repair equipment.  To stimulate technicians to modify their procedures to make equipment care easier. | Technical training material adapted from previous projects[4-6] and delivered by an expert biomedical engineer. Three-day training conducted at a central location for central engineers and at least one technician from each participating hospital.  Regular supervision visits (at least 3 monthly) for re-training, review and feedback, and to identify areas needing additional attention. |
| Procurement, installation, and maintenance structures  - procurement advice and support  - installation support  - maintenance procedures  - financing procedures | To enable reliable, continuous access to oxygen for all children and neonates.  To build hospital team capacity to maintain and scale-up oxygen systems.  To strengthen hospital’s technical capacity to maintain and repair equipment.  To make oxygen therapy affordable for both patients and hospitals. | Uniform equipment procurement led by coordination team, in collaboration with participating hospitals.  Equipment installation led by central technical team in collaboration with participating hospitals, and delivered in partnership with technicians from participating hospitals.  Equipment procedures and forms developed by coordination team in collaboration with participating hospitals. A comprehensive maintenance plan must involve the provider, installer, engineer, local technician, and local clinical staff (including timely access to technical support).  Local challenges identified during participatory planning, and addressed by local hospital teams.  Cost analysis conducted by coordination team with hospital-level data, and recommendations made for action by hospital administrators. |
| Infrastructure support  - improved power supply (e.g. solar power) | To ensure reliable, continuous access to oxygen for all children and neonates.  To make it easy to maintain oxygen equipment in good function. | Improved power system using solar capture and/or battery storage and/or generator back-up. The exact configuration was not been pre-specified, but will be based on hospital-level power evaluations, and recommendations from expert engineers/technicians (including mathematical modelling). Power system should be effective, efficient, user-friendly, and able to be maintained by local technicians.  Other potential needs may include: secure storage areas, enhanced security arrangements, workspace modification etc. |
| Strengthening health information systems  - clinical documentation  - medical records | To strengthen broader care processes.  To strengthen managerial support for oxygen therapy. | Support nursing and medical staff to improve documentation (e.g. adapt monitoring charts to include SpO_2_).  Support medical records staff with record keeping and reporting (e.g. basic electronic health reporting system on computer). |
| Strengthening quality improvement processes  - continuing education and morbidity review  - demonstrating quality improvement process  - quality improvement team building | To strengthen broader care processes.  To strengthen managerial support for oxygen therapy.  To build and sustain healthcare worker capacity and motivation to provide good clinical care.  To stimulate healthcare workers to make their work environment more conducive to good clinical care.  To build hospital team capacity to maintain and scale-up oxygen systems. | Support ongoing education and clinical review activities, including retraining as staff rotate.  Demonstrate behavioural and structural changes regarding oxygen therapy.  Encourage development, and support function, of multidisciplinary teams at the hospital level.  Embedded project nurse within each hospital to collect data, and support project activities. |

Equipment manufacturers: Airsep, Buffalo, United States of America (subsidiary of Chart Inc.); Lifebox Foundation, London, UK (<http://www.lifebox.org/>). We recommended the use of Airsep Newlife Elite concentrators and Lifebox pulse oximeters based on results of previous technical assessments[7-9], affordability, and field experience in resource-limited settings[10, 11]

**References**

1. WHO. Pocket Book of Hospital care for children: guidelines for the management of common childhood illnesses. 2nd ed. Geneva: World Health Organization; 2013.

2. WHO. Oxygen therapy for children. Geneva: World Health Organization; 2016.

3. Merrill MD. First Principles of Instruction. In: Reigeluth CM, Carr A, editors. Instructional Design Theories and Models: Building a Common Knowledge Base. III. New York: Routledge Publishers; 2009.

4. Gray AZ, Morpeth M. Oxygen Therapy Pilot Project, Lao PDR 2011-2013: Bringing affordable and life-saving oxygen to patients in district hospitals (Final Technical Report, September 2014). Centre for International Child Health, University of Melbourne, 2014.

5. Bradley BD, Light JD, Ebonyi AO, N'Jai PC, Ideh RC, Ebruke BE, et al. Implementation and 8-year follow-up of an uninterrupted oxygen supply system in a hospital in The Gambia. Int J Tuberc Lung Dis. 2016;20(8):1130-4. doi: 10.5588/ijtld.15.0889. PubMed PMID: 27393551.

6. Matai S, Peel D, Wandi F, Jonathan M, Subhi R, Duke T. Implementing an oxygen programme in hospitals in Papua New Guinea. Ann Trop Paediatr. 2008;28:71-8. doi: 10.1179/146532808X270716. PubMed PMID: 18318953.

7. Peel D, Neighbour R, Eltringham RJ. Evaluation of oxygen concentrators for use in countries with limited resources. Anaesthesia. 2013;68:706-12. doi: 10.1111/anae.12260. PubMed PMID: 23654218.

8. Lipnick MS, Feiner JR, Au P, Bernstein M, Bickler PE. The Accuracy of 6 Inexpensive Pulse Oximeters Not Cleared by the Food and Drug Administration: The Possible Global Public Health Implications. Anesth Analg. 2016;123(2):338-45. doi: 10.1213/ANE.0000000000001300. PubMed PMID: 27089002.

9. Dubowitz G, Breyer K, Lipnick M, Sall JW, Feiner J, Ikeda K, et al. Accuracy of the Lifebox pulse oximeter during hypoxia in healthy volunteers. Anaesthesia. 2013;68:1220-3. doi: 10.1111/anae.12382. PubMed PMID: 23992483.

10. Finch LC, Kim RY, Ttendo S, Kiwanuka JK, Walker Ia, Wilson IH, et al. Evaluation of a large-scale donation of Lifebox pulse oximeters to non-physician anaesthetists in Uganda. Anaesthesia. 2014;69:445-51. doi: 10.1111/anae.12632. PubMed PMID: 24738801.

11. Graham H, Tosif S, Gray A, Qazi S, Campbell H, Peel D, et al. Providing oxygen to children in hospitals: a realist review. Bull World Health Organ. 2017;95(4):288-302. doi: 10.2471/blt.16.186676.
