# Supplementary material for: Oxygen systems to improve clinical care and outcomes for children and neonates: A stepped-wedge cluster-randomised trial in Nigeria
Source: PLoS Med. 2019 Nov 11;16(11):e1002951. doi: 10.1371/journal.pmed.1002951 (PMC6844455; doi:10.1371/journal.pmed.1002951)
Supplement: S2 Table — (DOCX) [file pmed.1002951.s006.docx]

# **S2 Table – Timetable of project implementation**

*Paper: Graham HR, Bakare AA, Ayede AI, et al. Oxygen systems to improve clinical care and outcomes for children and neonates: a stepped-wedge cluster-randomised trial in Nigeria.*

This table describes the exact dates of implementation of the multi-faceted oxygen system intervention, with brief explanations of deviation from protocol. Colour shading indicates which components were done on schedule (green) or late (orange).

| **Sequence** | **Hospital** | **Pulse oximeters introduced** | **Oxygen system commissioned** | **Solar power commissioned** | **Clinical training conducted** | **Technician training conducted** |
| --- | --- | --- | --- | --- | --- | --- |
| 1 | Intended | November 2015 | March 2016 | | | Late 2016 |
|  | H1 | 30-Oct-2015 | 06-Apr-2016 | 23-Mar-2015 ^α^;  20-Oct-2016  29-Sep-2017 | 5-7-Apr-2016 | 5-9-Dec-2016 |
|  | H2 | 15-Oct-2015 | 13-Apr-2016 | 7-Apr-2016 ^α^;  19-Oct-2017 | 12-14-Apr-2016 | 5-9-Dec-2016 |
|  | H3 | 30-Oct-2015 | 08-Apr-2016 | 24-Mar-2015 ^α^;  29-Sep-2017 | 6-7-Apr-2016 | 5-9-Dec-2016 |
| 2 | Intended | November 2015 | July 2016 | | | Late 2016 |
|  | H4 | 28-Oct-2015 | 21-Jul-2016 | 16-Sep-2017 | 19-20-Jul-2016 | 5-9-Dec-2016 |
|  | H5 | 24-Nov-2015 | 05-Aug-2016^Ɏ^ | 07-Mar-2017 | 02-05-Aug-2016 ^Ɏ^ | 5-9-Dec-2016 |
|  | H6 | 20-Oct-2015 | 10-Jul-2016 | 14-Nov-2016 | 12-14-Jul-2016 | 5-9-Dec-2016 |
| 3 | Intended | November 2015 | November 2016 | | | Late 2016 |
|  | H7 | 28-Oct-2015 | 02-Nov-2016 | 19-Sep-2017 | 31-Oct-02-Nov-2016 | 5-9-Dec-2016 |
|  | H8 | 29-Oct-2015 | 17-Nov-2016 | 09-Mar-2017 | 15-17-Nov-2016 | 5-9-Dec-2016 |
|  | H9 | 15-Oct-2015 | 10-Nov-2016 | 19-Oct-2017 | 09-10-Nov-2016 | 5-9-Dec-2016 |
| 4 | Intended | November 2015 | March 2017 | | | Late 2016 |
|  | H10 | 09-Oct-2015 | 28-Mar-2017 | 28-Mar-2017 | 22-23-Mar-2016 | 5-9-Dec-2016 |
|  | H11 | 22-Oct-2015 | 16-Mar-2017 | 08-Nov-2017 | 15-16-Mar-2017 | 5-9-Dec-2016 |
|  | H12 | 14-Oct-2015 | 07-Mar-2017 | 28-Oct-2017 | 06-07-Mar-2017 | 5-9-Dec-2016 |

^α^ First solar power solution did not work, necessitating change of contractor and prototype design and testing.

^Ɏ^ Activities delayed till August because of health workers strike action.
