# Supplementary material for: Oxygen systems to improve clinical care and outcomes for children and neonates: A stepped-wedge cluster-randomised trial in Nigeria
Source: PLoS Med. 2019 Nov 11;16(11):e1002951. doi: 10.1371/journal.pmed.1002951 (PMC6844455; doi:10.1371/journal.pmed.1002951)
Supplement: S3 Table — (DOCX) [file pmed.1002951.s007.docx]

# **S3 Table – Primary Outcomes subgroup analyses**

*Paper: Graham HR, Bakare AA, Ayede AI, et al. Oxygen systems to improve clinical care and outcomes for children and neonates: a stepped-wedge cluster-randomised trial in Nigeria.*

These tables contain secondary analyses of child and neonatal mortality for particular subgroups: children and neonates with signs of hypoxaemia, children and neonates with SpO_2_<90%; children under 5 years of age (excluding neonates).

| \|  \|  \| **Mixed-model adjusted odds ratio (95% CI)** α \| \| \| \| \| \| \| \|  \| \| --- \| --- \| --- \| --- \| --- \| --- \| --- \| --- \| --- \| --- \| --- \| \|  \| **Deaths** \| **Primary analysisǂ** \| \| \| \| **Extended analysis§** \| \| \| \| **ICC**β \| \|  \| **n/N (%)*** \| **Basic model** \| \| **severity-adjusted** \| \| **Basic model** \| \| **severity-adjusted** \| \| **(95%CI)** \| \| **A – Signs of hypoxaemia** \| \|  \| **P** \|  \| **P** \|  \| **P** \|  \| **P** \|  \| \| Child   - Pre-intervention - Pulse oximetry - Full O_2_ system \| 295/4202 (7.0)  141/1740 (7.5)  273/2255 (12.1) \| -  0.82 (0.55-1.24) \| 0.351 \| -  0.85 (0.54-1.32) \| 0.469 \| -  1.94 (1.09-3.45)  1.30 (0.85-2.00) \| 0.024  0.224 \| -  1.39 (0.75-2.57)  1.07 (0.68-1.69) \| 0.299  0.759 \| 0.10  (0.10-0.21) \| \| Child ALRI   - Pre-intervention - Pulse oximetry - Full O_2_ system \| 96/1044 (9.2)  39/451 (8.7)  88/643 (13.7) \| -  0.98 (0.422-2.28) \| 0.965 \| -  1.12 (0.47-2.66) \| 0.798 \| -  0.56 (0.20-1.58)  0.60 (0.31-1.16) \| 0.275  0.132 \| -  0.42 (0.14-1.30)  0.51 (0.25-1.04) \| 0.133  0.064 \| 0.11  (0.05-0.26) \| \| Neonate   - Pre-intervention - Pulse oximetry - Full O_2_ system \| 366/1949 (18.8~~)~~  125/658 (19.0)  281/1129 (24.9) \| -  1.46 (0.95-2.24) \| 0.087 \| -  1.89 (1.17-3.07) \| 0.010 \| -  1.07 (0.59-1.94)  1.09 (0.70-1.70) \| 0.826  0.691 \| -  0.72 (0.37-1.39)  0.82 (0.50-1.34) \| 0.327  0.430 \| 0.06  (0.02-0.16) \| \| Preterm/LBW neonate   - Pre-intervention - Pulse oximetry - Full O_2_ system \| 138/582 (23.7)  47/202 (23.3)  101/295 (34.2) \| -  1.66 (0.81-3.44) \| 0.169 \| -  2.35 (1.05-5.26) \| 0.038 \| -  0.89 (0.34-2.36)  0.99 (0.50-1.98) \| 0.819  0.982 \| -  0.57 (0.20-1.66)  0.69 (0.32-1.48) \| 0.304  0.340 \| 0.04  (0.01-0.19) \|  \| **B – SpO_2_<90%** \|  \|  \|  \|  \|  \|  \|  \|  \|  \|  \| \| --- \| --- \| --- \| --- \| --- \| --- \| --- \| --- \| --- \| --- \| --- \| \| Child   - Pre-intervention - Pulse oximetry - Full O_2_ system \| *18/76 (23.7)*  108/530 (20.4)  265/1227 (21.6) \| -  0.95 (0.60-1.49) \| 0.818 \| -  0.95 (0.58-1.58) \| 0.852 \|  \|  \|  \|  \| 0.02  (0.01-0.08) \| \| Child ALRI   - Pre-intervention - Pulse oximetry - Full O_2_ system \| *3/23 (13.0)*  32/182 (17.6)  75/428 (17.5) \| -  0.93 (0.35-2.51) \| 0.889 \| -  1.32 (0.46-3.80) \| 0.613 \|  \|  \|  \|  \| 0.14  (0.04-0.38) \| \| Neonate   - Pre-intervention - Pulse oximetry - Full O_2_ system \| *20/53 (37.7~~)~~*  121/485 (25.0)  369/1231 (30.0) \| -  1.53 (1.00-2.36) \| 0.053 \| -  1.83 (1.14-2.94) \| 0.013 \|  \|  \|  \|  \| 0.03  (0.01-0.12) \| \| Preterm/LBW neonate   - Pre-intervention - Pulse oximetry - Full O_2_ system \| *6/9 (66.7)*  48/153 (31.4)  150/372 (40.3) \| -  1.43 (0.66-3.09) \| 0.360 \| -  1.94 (0.86-4.35) \| 0.110 \|  \|  \|  \|  \| 0.04  (0.01-0.19) \|   *Denominators vary according to the population included. ǂ Primary analysis compares Full Oxygen system and Pulse Oximetry periods. § Extended analysis compares Pulse Oximetry and Full Oxygen system periods to the Pre-intervention period. α Mixed-model odds ratios account for the clustering of patients within hospitals and adjust for time trends. Under the stepped-wedge design, the adjusted odds ratios are calculated with the use of all data points in the intervention period versus the comparison period and therefore represent the average odds of exposure to the intervention. β Intra-cluster correlation coefficient (ICC). γ Child under 15 years of age, excluding neonates (<28 days of age). ALRI = cough or difficult breathing and any of: fast breathing, lower chest wall indrawing. CI = confidence interval. LBW = low birth weight, <2500 grams. Preterm, defined as <37 weeks gestational age. Signs of hypoxaemia included: severe respiratory distress; central cyanosis; altered conscious state; respiratory rate >70 per minute; unable to feed due to fast breathing.  **Effect of the intervention(s) on mortality, showing the primary analysis (full oxygen period versus pulse oximetry period) and extended analysis (comparing pulse oximetry and full oxygen system periods to the pre-intervention period). Restricted to patients with signs of hypoxaemia (A) or SpO_2_<90% (B).** |
| --- | --- | --- | --- | --- | --- | --- | --- | --- | --- | --- | --- | --- | --- | --- | --- | --- | --- | --- | --- | --- | --- | --- | --- | --- | --- | --- | --- | --- | --- | --- | --- | --- | --- | --- | --- | --- | --- | --- | --- | --- | --- | --- | --- | --- | --- | --- | --- | --- | --- | --- | --- | --- | --- | --- | --- | --- | --- | --- | --- | --- | --- | --- | --- | --- | --- | --- | --- | --- | --- | --- | --- | --- | --- | --- | --- | --- | --- | --- | --- | --- | --- | --- | --- | --- | --- | --- | --- | --- | --- | --- | --- | --- | --- | --- | --- | --- | --- | --- | --- | --- | --- | --- | --- | --- | --- | --- | --- | --- | --- | --- | --- | --- | --- | --- | --- | --- | --- | --- | --- | --- | --- | --- | --- | --- | --- | --- | --- | --- | --- | --- | --- | --- | --- | --- | --- | --- | --- | --- | --- | --- | --- | --- | --- |

| \|  \|  \| **Mixed-model adjusted odds ratio (95% CI)** α \| \| \| \| \| \| \| \|  \| \| --- \| --- \| --- \| --- \| --- \| --- \| --- \| --- \| --- \| --- \| --- \| \|  \| **Deaths** \| **Primary analysisǂ** \| \| \| \| **Extended analysis§** \| \| \| \| **ICC**β \| \|  \| **n/N (%)*** \| **Basic model** \| \| **severity-adjusted** \| \| **Basic model** \| \| **severity-adjusted** \| \| **(95%CI)** \| \| **Children under 5 years of age (excluding neonates)** \| \|  \| **P** \|  \| **P** \|  \| **P** \|  \| **P** \|  \| \| Child   - Pre-intervention - Pulse oximetry - Full O_2_ system \| 511/12559 (4.1)  187/5145 (3.6)  342/6883 (5.0) \| -  1.08 (0.75-1.54) \| 0.691 \| -  1.16 (0.78-1.74) \| 0.457 \| -  1.25 (0.78-2.01)  1.16 (0.83-1.63) \| 0.352  0.383 \| -  1.12 (0.65-1.94)  1.18 (0.80-1.76) \| 0.673  0.406 \| 0.05  (0.02-0.10) \| \| Child ALRI   - Pre-intervention - Pulse oximetry - Full O_2_ system \| 108/1786 (6.1)  41/689 (6.0)  87/1072 (8.1) \| -  1.00 (0.43-2.29) \| 0.997 \| -  1.32 (0.54-3.20) \| 0.544 \| -  0.45 (0.17-1.22)  0.51 (0.27-0.95) \| 0.117  0.033 \| -  0.33 (0.11-0.99)  0.46 (0.23-0.92) \| 0.133  0.027 \| 0.10  (0.04-0.23) \|   *Denominators vary according to the population included. ǂ Primary analysis compares Full Oxygen system and Pulse Oximetry periods. § Extended analysis compares Pulse Oximetry and Full Oxygen system periods to the Pre-intervention period. α Mixed-model odds ratios account for the clustering of patients within hospitals and adjust for time trends. Under the stepped-wedge design, the adjusted odds ratios are calculated with the use of all data points in the intervention period versus the comparison period and therefore represent the average odds of exposure to the intervention. β Intra-cluster correlation coefficient (ICC).. ALRI = cough or difficult breathing and any of: fast breathing, lower chest wall indrawing. CI = confidence interval  **Effect of the intervention(s) on mortality, showing the primary analysis (full oxygen period versus pulse oximetry period) and extended analysis (comparing pulse oximetry and full oxygen system periods to the pre-intervention period). Restricted to patients under the age of 5 years (excluding neonates).** |
| --- | --- | --- | --- | --- | --- | --- | --- | --- | --- | --- | --- | --- | --- | --- | --- | --- | --- | --- | --- | --- | --- | --- | --- | --- | --- | --- | --- | --- | --- | --- | --- | --- | --- | --- | --- | --- | --- | --- | --- | --- | --- | --- | --- | --- | --- | --- | --- | --- | --- | --- | --- | --- | --- | --- | --- | --- | --- | --- | --- | --- | --- | --- | --- | --- | --- | --- |
