# Supplementary material for: Oxygen systems to improve clinical care and outcomes for children and neonates: A stepped-wedge cluster-randomised trial in Nigeria
Source: PLoS Med. 2019 Nov 11;16(11):e1002951. doi: 10.1371/journal.pmed.1002951 (PMC6844455; doi:10.1371/journal.pmed.1002951)
Supplement: S4 Table — (DOCX) [file pmed.1002951.s008.docx]

# **S4 Table – Primary clinical outcomes by individual hospital**

*Paper: Graham HR, Bakare AA, Ayede AI, et al. Oxygen systems to improve clinical care and outcomes for children and neonates: a stepped-wedge cluster-randomised trial in Nigeria.*

Effect of the intervention(s) on primary clinical outcomes for children, children with ALRI, neonates, and preterm neonates, showing the primary analysis (full oxygen period versus pulse oximetry period) and extended analysis (comparing pulse oximetry and full oxygen system periods to the pre-intervention period). By individual hospital.

| \|  \|  \|  \| **Odds ratio (95% CI) α** \| \| \| \| \| --- \| --- \| --- \| --- \| --- \| --- \| --- \| \|  \|  \|  \| **Primary analysisǂ** \| \| **Extended analysis§** \| \| \| **Child** \| \| **Deaths**  **n/N (%)*** \| **Basic model** \| **severity-adjusted** \| **Basic model** \| **severity-adjusted** \| \| **H1** \| Child \|  \|  \|  \|  \|  \| \|  \| -   Pre-intervention \| 130/1865 (7.0%) \|  \|  \| Ref \| Ref \| \|  \| -   Pulse oximetry \| 25/316 (7.9%) \| Ref \| Ref \| 1.15 (0.73-1.79) \| 1.59 (0.90-2.79) \| \|  \| -   Full O2 system \| 66/1452 (4.5%) \| 0.55 (0.34-0.89) \| 0.46 (0.25-0.85) \| 0.64 (0.47-0.86) \| 0.78 (0.54-1.12) \| \| **H2** \| Child \|  \|  \|  \|  \|  \| \|  \| -   Pre-intervention \| 51/705 (7.2%) \|  \|  \| Ref \| Ref \| \|  \| -   Pulse oximetry \| 8/123 (6.5%) \| Ref \| Ref \| 0.89 (0.41-1.93) \| 1.24 (0.54-2.86) \| \|  \| -   Full O2 system \| 25/420 (6.0%) \| 0.91 (0.40-2.07) \| 0.50 (0.19-1.32) \| 0.81 (0.49-1.33) \| 0.82 (0.46-1.45) \| \| **H3** \| Child \|  \|  \|  \|  \|  \| \|  \| -   Pre-intervention \| 88/1362 (6.5%) \|  \|  \| Ref \| Ref \| \|  \| -   Pulse oximetry \| 23/287 (8.0%) \| Ref \| Ref \| 1.26 (0.78-2.03) \| 1.31 (0.74-2.29) \| \|  \| -   Full O2 system \| 90/1217 (7.4%) \| 0.92 (0.57-1.48) \| 0.77 (0.44-1.35) \| 1.16 (0.85-1.57) \| 1.08 (0.74-1.55) \| \| **H4** \| Child \|  \|  \|  \|  \|  \| \|  \| -   Pre-intervention \| 79/1897 (4.2%) \|  \|  \| Ref \| Ref \| \|  \| -   Pulse oximetry \| 22/541 (4.1%) \| Ref \| Ref \| 0.98 (0.60-1.58) \| 1.07 (0.63-1.84) \| \|  \| -   Full O2 system \| 44/1054 (4.2%) \| 1.03 (0.61-1.73) \| 1.04 (0.59-1.84) \| 1.00 (0.69-1.46) \| 1.16 (0.75-1.79) \| \| **H5** \| Child \|  \|  \|  \|  \|  \| \|  \| -   Pre-intervention \| 28/1186 (2.4%) \|  \|  \| Ref \| Ref \| \|  \| -   Pulse oximetry \| 12/518 (2.3%) \| Ref \| Ref \| 0.98 (0.49-1.94) \| 1.90 (0.88-4.10) \| \|  \| -   Full O2 system \| 40/1151 (3.5%) \| 1.52 (0.79-2.92) \| 1.23 (0.61-2.51) \| 1.49 (0.91-2.4) \| 2.54 (1.38-4.66) \| \| **H6** \| Child \|  \|  \|  \|  \|  \| \|  \| -   Pre-intervention \| 29/468 (6.2%) \|  \|  \| Ref \| Ref \| \|  \| -   Pulse oximetry \| 10/163 (6.1%) \| Ref \| Ref \| 0.99 (0.47-2.08) \| 0.50 (0.21-1.22) \| \|  \| -   Full O2 system \| 11/328 (3.4%) \| 0.53 (0.22-1.28) \| 0.71 (0.25-1.97) \| 0.53 (0.26-1.07) \| 0.30 (0.13-0.74) \| \| **H7** \| Child \|  \|  \|  \|  \|  \| \|  \| -   Pre-intervention \| 49/2864 (1.7%) \|  \|  \| Ref \| Ref \| \|  \| -   Pulse oximetry \| 27/1480 (1.8%) \| Ref \| Ref \| 1.07 (0.66-1.71) \| 0.67 (0.34-1.33) \| \|  \| -   Full O2 system \| 36/1157 (3.1%) \| 1.73 (1.04-2.86) \| 2.86 (1.42-5.73) \| 1.84 (1.19-2.85) \| 2.01 (1.13-3.58) \| \| **H8** \| Child \|  \|  \|  \|  \|  \| \|  \| -   Pre-intervention \| 32/965 (3.3%) \|  \|  \| Ref \| Ref \| \|  \| -   Pulse oximetry \| 27/631 (4.3%) \| Ref \| Ref \| 1.30 (0.77-2.20) \| 1.16 (0.65-2.08) \| \|  \| -   Full O2 system \| 37/614 (6.0%) \| 1.43 (0.86-2.39) \| 1.86 (1.05-3.30) \| 1.87 (1.15-3.03) \| 2.18 (1.28-3.69) \| \| **H9** \| Child \|  \|  \|  \|  \|  \| \|  \| -   Pre-intervention \| 4/244 (1.6%) \|  \|  \| Ref \| Ref \| \|  \| -   Pulse oximetry \| 5/149 (3.4%) \| Ref \| Ref \| 2.08 (0.55-7.88) \| 2.96 (0.65-13.60) \| \|  \| -   Full O2 system \| 11/324 (3.4%) \| 1.01 (0.35-2.97) \| 1.05 (0.0.28-3.98) \| 2.11 (0.66-6.70) \| 3.59 (0.95-13.59) \| \| **H10** \| Child \|  \|  \|  \|  \|  \| \|  \| -   Pre-intervention \| 105/2444 (4.3%) \|  \|  \| Ref \| Ref \| \|  \| -   Pulse oximetry \| 40/1243 (3.2%) \| Ref \| Ref \| 0.74 (0.51-1.07) \| 0.91 (0.60-1.38) \| \|  \| -   Full O2 system \| 40/794 (5.0%) \| 1.60 (1.02-2.50) \| 1.36 (0.81-2.27) \| 1.18 (0.81-1.72) \| 1.17 (0.76-1.81) \| \| **H11** \| Child \|  \|  \|  \|  \|  \| \|  \| -   Pre-intervention \| 29/558 (5.2%) \|  \|  \| Ref \| Ref \| \|  \| -   Pulse oximetry \| 16/474 (3.4%) \| Ref \| Ref \| 0.64 (0.34-1.19) \| 0.75 (0.36-1.55) \| \|  \| -   Full O2 system \| 11/311 (3.5%) \| 1.05 (0.48-2.29) \| 0.77 (0.29-2.03) \| 0.67 (0.33-1.36) \| 0.66 (0.28-1.54) \| \| **H12** \| Child \|  \|  \|  \|  \|  \| \|  \| -   Pre-intervention \| 8/509 (1.6%) \|  \|  \| Ref \| Ref \| \|  \| -   Pulse oximetry \| 12/515 (2.3%) \| Ref \| Ref \| 1.49 (0.61-3.69) \| 3.21 (0.68-15.01) \| \|  \| -   Full O2 system \| 11/381 (2.9%) \| 1.25 (0.54-2.86) \| 1.47 (0.60-3.57) \| 1.86 (0.74-4.67) \| 4.70 (1.00-22.04) \| \|  \|  \|  \| **Odds ratio (95% CI) α** \| \| \| \| \|  \|  \|  \| **Primary analysisǂ** \| \| **Extended analysis§** \| \| \| **Child ALRI** \| \| **Deaths**  **n/N (%)*** \| **Basic model** \| **severity-adjusted** \| **Basic model** \| **severity-adjusted** \| \| **H1** \| Child ALRI \|  \|  \|  \|  \|  \| \|  \| -   Pre-intervention \| 18/277 (6.5%) \|  \|  \| - \| - \| \|  \| -   Pulse oximetry \| 5/39 (12.8%) \| - \| - \| 2.12 (0.74-6.07) \| 2.04 (0.63-6.62) \| \|  \| -   Full O2 system \| 15/250 (6.0%) \| 0.43 (0.15-1.27) \| 0.28 (0.07-1.12) \| 0.92 (0.45-1.86) \| 0.71 (0.31-1.61) \| \| **H2** \| Child ALRI \|  \|  \|  \|  \|  \| \|  \| -   Pre-intervention \| 18/93 (19.4%) \|  \|  \| - \| - \| \|  \| -   Pulse oximetry \| 6/20 (30.0%) \| - \| - \| 1.79 (0.60-5.29) \| 3.08 (0.89-10.73) \| \|  \| -   Full O2 system \| 8/58 (13.8%) \| 0.37 (0.11-1.26) \| 0.14 (0.03-0.81) \| 0.67 (0.27-1.65) \| 0.97 (0.34-2.80) \| \| **H3** \| Child ALRI \|  \|  \|  \|  \|  \| \|  \| -   Pre-intervention \| 13/158 (8.2%) \|  \|  \| - \| - \| \|  \| -   Pulse oximetry \| 3/35 (8.6%) \| - \| - \| 1.05 (0.28-3.88) \| 1.24 (0.28-5.44) \| \|  \| -   Full O2 system \| 29/169 (17.2%) \| 2.21 (0.63-7.71) \| 2.53 (0.50-12.90) \| 2.31 (1.15-4.63) \| 2.61 (1.14-5.98) \| \| **H4** \| Child ALRI \|  \|  \|  \|  \|  \| \|  \| -   Pre-intervention \| 16/287 (5.6%) \|  \|  \| - \| - \| \|  \| -   Pulse oximetry \| 6/85 (7.1%) \| - \| - \| 1.29 (0.49-3.40) \| 1.07 (0.34-3.33) \| \|  \| -   Full O2 system \| 11/146 (7.5%) \| 1.07 (0.38-3.01) \| 0.96 (0.30-3.05) \| 1.38 (0.62-3.06) \| 1.51 (0.60-3.79) \| \| **H5** \| Child ALRI \|  \|  \|  \|  \|  \| \|  \| -   Pre-intervention \| 7/168 (4.2%) \|  \|  \| - \| - \| \|  \| -   Pulse oximetry \| 4/75 (5.3%) \| - \| - \| 1.30 (0.37-4.57) \| 1.59 (0.29-8.81) \| \|  \| -   Full O2 system \| 13/163 (8.0%) \| 1.54 (0.48-4.89) \| 2.54 (0.56-11.62) \| 1.99 (0.37-4.57) \| 4.79 (1.11-20.68) \| \| **H6** \| Child ALRI \|  \|  \|  \|  \|  \| \|  \| -   Pre-intervention \| 4/49 (8.2%) \|  \|  \| - \| - \| \|  \| -   Pulse oximetry \| 2/24 (8.3%) \| - \| - \| 1.02 (0.17-6.02) \| 0.36 (0.01-10.76) \| \|  \| -   Full O2 system \| 2/24 (8.3%) \| 1.00 (0.13-7.75) \| unable to compute \| 1.02 (0.17-6.02) \| 0.03 (0.00-4.13) \| \| **H7** \| Child ALRI \|  \|  \|  \|  \|  \| \|  \| -   Pre-intervention \| 11/385 (2.9%) \|  \|  \| - \| - \| \|  \| -   Pulse oximetry \| 2/189 (1.1%) \|  \|  \| 0.36 (0.08-1.66) \| 0.19 (0.03-1.21) \| \|  \| -   Full O2 system \| 0/151 (0.0%) \| unable to compute \| unable to compute \| unable to compute \| unable to compute \| \| **H8** \| Child ALRI \|  \|  \|  \|  \|  \| \|  \| -   Pre-intervention \| 7/131 (5.3%) \|  \|  \| - \| - \| \|  \| -   Pulse oximetry \| 4/84 (4.8%) \| - \| - \| 0.89 (0.25-3.12) \| 0.98 (0.24-3.99) \| \|  \| -   Full O2 system \| 5/75 (6.7%) \| 1.43 (0.37-5.53) \| 7.17 (0.74-69.79) \| 1.27 (0.39-4.14) \| 1.75 (0.43-7.18) \| \| **H9** \| Child ALRI \|  \|  \|  \|  \|  \| \|  \| -   Pre-intervention \| 0/12 (0.0%) \|  \|  \|  \|  \| \|  \| -   Pulse oximetry \| 2/7 (28.6%) \| - \|  \|  \|  \| \|  \| -   Full O2 system \| 3/10 (30.0%) \| 1.07 (0.13-8.98) \| unable to compute \| unable to compute \| unable to compute \| \| **H10** \| Child ALRI \|  \|  \|  \|  \|  \| \|  \| -   Pre-intervention \| 18/254 (7.1%) \|  \|  \| - \| - \| \|  \| -   Pulse oximetry \| 6/156 (3.8%) \| - \| - \| 0.52 (0.20-1.35) \| 0.56 (0.20-1.59) \| \|  \| -   Full O2 system \| 7/101 (6.9%) \| 1.86 (0.61-5.71) \| 1.47 (0.37-5.80) \| 0.98 (0.39-2.41) \| 0.80 (0.28-2.33) \| \| **H11** \| Child ALRI \|  \|  \|  \|  \|  \| \|  \| -   Pre-intervention \| 4/58 (6.9%) \|  \|  \| - \| - \| \|  \| -   Pulse oximetry \| 3/23 (13.0%) \| - \| - \| 2.03 (0.42-9.86) \| 3.53 (0.12-101.35) \| \|  \| -   Full O2 system \| 3/24 (12.5%) \| 0.95 (0.17-5.28) \| 0.18 (0.00-7.80) \| 1.93 (0.40-9.36) \| 3.86 (0.22-67.11) \| \| **H12** \| Child ALRI \|  \|  \|  \|  \|  \| \|  \| -   Pre-intervention \| 0/15 (0%) \|  \|  \|  \|  \| \|  \| -   Pulse oximetry \| 1/22 (4.5%) \| - \|  \|  \|  \| \|  \| -   Full O2 system \| 1/11 (9.1%) \| 2.1 (0.12-37.12) \| unable to compute \| unable to compute \| unable to compute \| \|  \|  \|  \| **Odds ratio (95% CI) α** \| \| \| \| \|  \|  \|  \| **Primary analysisǂ** \| \| **Extended analysis§** \| \| \| **Neonate** \| \| **Deaths**  **n/N (%)*** \| **Basic model** \| **severity-adjusted** \| **Basic model** \| **severity-adjusted** \| \| **H1** \| Neonate \|  \|  \|  \|  \|  \| \|  \| -   Pre-intervention \| 396/2320 (17.1%) \|  \|  \| - \| - \| \|  \| -   Pulse oximetry \| 36/303 (11.9%) \| - \| - \| 0.66 (0.46-0.94) \| 0.65 (0.43-0.98) \| \|  \| -   Full O2 system \| 250/1769 (14.1%) \| 1.22 (0.84-1.77) \| 1.00 (0.0.66-1.53) \| 0.80 (0.67-0.95) \| 0.67 (0.55-0.82) \| \| **H2** \| Neonate \|  \|  \|  \|  \|  \| \|  \| -   Pre-intervention \| 18/86 (20.9%) \|  \|  \| - \| - \| \|  \| -   Pulse oximetry \| 5/15 (33.3%) \| - \| - \| 1.89 (0.57-6.23) \| 5.50 (0.89-33.94) \| \|  \| -   Full O2 system \| 18/79 (22.8%) \| 0.59 (0.18-1.95) \| 0.34 (0.07-1.79) \| 1.11 (0.53-2.33) \| 1.05 (0.38-2.94) \| \| **H3** \| Neonate \|  \|  \|  \|  \|  \| \|  \| -   Pre-intervention \| 31/131 (23.7%) \|  \|  \| - \| - \| \|  \| -   Pulse oximetry \| 6/31 (19.4%) \| - \| - \| 0.77 (0.29-2.06) \| 1.19 (0.36-3.93) \| \|  \| -   Full O2 system \| 11/80 (13.8%) \| 0.66 (0.22-1.99) \| 0.48 (0.11-2.05) \| 0.51 (0.24-1.09) \| 0.55 (0.19-1.54) \| \| **H4** \| Neonate \|  \|  \|  \|  \|  \| \|  \| -   Pre-intervention \| 131/1776 (7.4%) \|  \|  \| - \| - \| \|  \| -   Pulse oximetry \| 25/393 (6.4%) \| - \| - \| 0.85 (0.55-1.33) \| 0.70 (0.42-1.16) \| \|  \| -   Full O2 system \| 67/874 (7.7%) \| 1.22 (0.76-1.97) \| 1.38 (0.80-2.39) \| 1.04 (0.77-1.42) \| 0.97 (0.69-1.38) \| \| **H5** \| Neonate \|  \|  \|  \|  \|  \| \|  \| -   Pre-intervention \| 21/254 (8.3%) \|  \|  \| - \| - \| \|  \| -   Pulse oximetry \| 3/118 (2.5%) \| - \| - \| 0.29 (0.08-0.99) \| 0.37 (0.09-1.43) \| \|  \| -   Full O2 system \| 14/257 (5.4%) \| 2.21 (0.62-7.84) \| 2.72 (0.68-10.92) \| 0.64 (0.32-1.29) \| 1.03 (0.43-2.47) \| \| **H6** \| Neonate \|  \|  \|  \|  \|  \| \|  \| -   Pre-intervention \| 29/209 (13.9%) \|  \|  \| - \| - \| \|  \| -   Pulse oximetry \| 8/42 (19.0%) \| - \| - \| 1.46 (0.62-3.47) \| 0.50 (0.15-1.68) \| \|  \| -   Full O2 system \| 13/50 (26.0%) \| 1.49 (0.55-4.04) \| 2.12 (0.45-9.99) \| 2.18 (1.04-4.59) \| 1.52 (0.56-4.12) \| \| **H7** \| Neonate \|  \|  \|  \|  \|  \| \|  \| -   Pre-intervention \| 169/1998 (8.5%) \|  \|  \| - \| - \| \|  \| -   Pulse oximetry \| 71/799 (8.9%) \| - \| - \| 1.06 (0.79-1.41) \| 3.41 (1.32-8.83) \| \|  \| -   Full O2 system \| 58/572 (10.1%) \| 1.16 (0.80-1.67) \| 1.34 (0.88-2.04) \| 1.22 (0.89-1.67) \| 6.56 (2.64-16.32) \| \| **H8** \| Neonate \|  \|  \|  \|  \|  \| \|  \| -   Pre-intervention \| 48/552 (8.7%) \|  \|  \| - \| - \| \|  \| -   Pulse oximetry \| 21/211 (10.0%) \| - \| - \| 1.16 (0.68-1.99) \| 1.24 (0.52-2.95) \| \|  \| -   Full O2 system \| 27/214 (12.6%) \| 1.31 (0.71-2.39) \| 1.16 (0.52-2.59) \| 1.52 (0.92-2.50) \| 2.51 (0.93-6.72) \| \| **H9** \| Neonate \|  \|  \|  \|  \|  \| \|  \| -   Pre-intervention \| 0/3 (0%) \|  \|  \|  \|  \| \|  \| -   Pulse oximetry \| 0/5 (0%) \|  \|  \|  \|  \| \|  \| -   Full O2 system \| 0/2 (0%) \| unable to compute \| unable to compute \| unable to compute \| unable to compute \| \| **H10** \| Neonate \|  \|  \|  \|  \|  \| \|  \| -   Pre-intervention \| 70/1369 (5.1%) \|  \|  \| - \| - \| \|  \| -   Pulse oximetry \| 28/663 (4.2%) \| - \| - \| 0.82 (0.52-1.28) \| 0.62 (0.29-1.32) \| \|  \| -   Full O2 system \| 28/425 (6.6%) \| 1.60 (0.93-2.74) \| 1.46 (0.77-2.76) \| 1.31 (0.83-2.06) \| 1.02 (0.42-2.48) \| \| **H11** \| Neonate \|  \|  \|  \|  \|  \| \|  \| -   Pre-intervention \| 0/46 (0%) \|  \|  \|  \|  \| \|  \| -   Pulse oximetry \| 1/47 (2.1%) \|  \|  \|  \|  \| \|  \| -   Full O2 system \| 0/38 (0%) \| unable to compute \| unable to compute \| unable to compute \| unable to compute \| \| **H12** \| Neonate \|  \|  \|  \|  \|  \| \|  \| -   Pre-intervention \| 0/1 (0%) \|  \|  \|  \|  \| \|  \| -   Pulse oximetry \| 0/0 \|  \|  \|  \|  \| \|  \| -   Full O2 system \| 0/0 \| unable to compute \| unable to compute \| unable to compute \| unable to compute \| \|  \|  \|  \| **Odds ratio (95% CI) α** \| \| \| \| \|  \|  \|  \| **Primary analysisǂ** \| \| **Extended analysis§** \| \| \| **Preterm/LBW** \| \| **Deaths**  **n/N (%)*** \| **Basic model** \| **severity-adjusted** \| **Basic model** \| **severity-adjusted** \| \| **H1** \| Preterm/LBW \|  \|  \|  \|  \|  \| \|  \| -   Pre-intervention \| 205/704 (29.1%) \|  \|  \| - \| - \| \|  \| -   Pulse oximetry \| 14/89 (15.7%) \| - \|  \| 0.45 (0.25-0.82) \| 0.57 (0.30-1.07) \| \|  \| -   Full O2 system \| 123/470 (26.2%) \| 1.90 (1.04-3.48) \| 1.46 (0.75-2.82) \| 0.86 (0.66-1.12) \| 0.85 (0.63-1.13) \| \| **H2** \| Preterm/LBW \|  \|  \|  \|  \|  \| \|  \| -   Pre-intervention \| 3/13 (23.1%) \|  \|  \| - \| - \| \|  \| -   Pulse oximetry \| 1/1 (100.0%) \|  \|  \| unable to compute \| unable to compute \| \|  \| -   Full O2 system \| 8/19 (42.1%) \| unable to compute \| unable to compute \| 2.42 (0.50-11.76) \| 2.09 (0.37-11.70) \| \| **H3** \| Preterm/LBW \|  \|  \|  \|  \|  \| \|  \| -   Pre-intervention \| 3/12 (25.0%) \|  \|  \| - \| - \| \|  \| -   Pulse oximetry \| 3/17 (17.7%) \| - \|  \| 0.64 (0.11-3.91) \| 5.96 (0.27-132.81) \| \|  \| -   Full O2 system \| 2/21 (9.5%) \| 0.49 (0.073.34) \| 0.15 (0.01-2.02) \| 0.32 (0.04-2.24) \| 1.67 (0.09-30.78) \| \| **H4** \| Preterm/LBW \|  \|  \|  \|  \|  \| \|  \| -   Pre-intervention \| 63/320 (19.7%) \|  \|  \| - \| - \| \|  \| -   Pulse oximetry \| 8/65 (12.3%) \| - \| - \| 0.57 (0.26-1.26) \| 0.57 (0.24-1.32) \| \|  \| -   Full O2 system \| 30/203 (14.8%) \| 1.24 (0.54-2.85) \| 1.72 (0.68-4.40) \| 0.71 (0.44-1.14) \| 0.84 (0.50-1.41) \| \| **H5** \| Preterm/LBW \|  \|  \|  \|  \|  \| \|  \| -   Pre-intervention \| 2/30 (6.7%) \|  \|  \| - \| -- \| \|  \| -   Pulse oximetry \| 2/8 (25.0%) \|  \|  \| 4.67 (0.54-4.03) \| unable to compute \| \|  \| -   Full O2 system \| 0/12 (0.0%) \| unable to compute \| unable to compute \| unable to compute \| unable to compute \| \| **H6** \| Preterm/LBW \|  \|  \|  \|  \|  \| \|  \| -   Pre-intervention \| 8/46 (17.4%) \|  \|  \| - \| - \| \|  \| -   Pulse oximetry \| 0/10 (0.0%) \|  \|  \| unable to compute \| unable to compute \| \|  \| -   Full O2 system \| 2/12 (16.7%) \| unable to compute \| unable to compute \| 0.95 (0.17-5.19) \| 8.83 (0.54-144.17) \| \| **H7** \| Preterm/LBW \|  \|  \|  \|  \|  \| \|  \| -   Pre-intervention \| 16/367 (4.4%) \|  \|  \| - \| - \| \|  \| -   Pulse oximetry \| 39/207 (18.8%) \| - \|  \| 5.09 (2.77-9.37) \| 5.22 (2.52-10.78) \| \|  \| -   Full O2 system \| 24/151 (15.9%) \| 0.81 (0.47-1.42) \| 1.07 (0.57-2.02) \| 4.15 (2.13-8.06) \| 5.60 (2.64-11.88) \| \| **H8** \| Preterm/LBW \|  \|  \|  \|  \|  \| \|  \| -   Pre-intervention \| 8/125 (6.4%) \|  \|  \| - \| - \| \|  \| -   Pulse oximetry \| 6/67 (9.0%) \| - \| - \| 1.44 (0.48-4.33) \| 1.11 (0.28-4.43) \| \|  \| -   Full O2 system \| 4/49 (8.2%) \| 0.90 (0.24-3.39) \| 1.25 (0.23-6.71) \| 1.30 (0.37-4.53) \| 1.48 (0.33-6.63) \| \| **H9** \| Preterm/LBW \|  \|  \|  \|  \|  \| \|  \| -   Pre-intervention \| 0/2 (0.0%) \|  \|  \|  \|  \| \|  \| -   Pulse oximetry \| 0/0 \|  \|  \|  \|  \| \|  \| -   Full O2 system \| 0/0 \| unable to compute \| unable to compute \| unable to compute \| unable to compute \| \| **H10** \| Preterm/LBW \|  \|  \|  \|  \|  \| \|  \| -   Pre-intervention \| 18/257 (7.0%) \|  \|  \| - \| - \| \|  \| -   Pulse oximetry \| 8/141 (5.7%) \| - \|  \| 0.80 (0.34-1.89) \| 1.09 (0.43-2.76) \| \|  \| -   Full O2 system \| 10/96 (10.4%) \| 1.93 (0.73-5.09) \| 1.93 (0.60-6.15) \| 1.54 (0.69-3.48) \| 2.00 (0.79-5.11) \| \| **H11** \| Preterm/LBW \|  \|  \|  \|  \|  \| \|  \| -   Pre-intervention \| 0/2 (0.0%) \|  \|  \|  \|  \| \|  \| -   Pulse oximetry \| 1/4 (25.0%) \|  \|  \|  \|  \| \|  \| -   Full O2 system \| 0/9 (0.0%) \| unable to compute \| unable to compute \| unable to compute \| unable to compute \| \| **H12** \| Preterm/LBW \|  \|  \|  \|  \|  \| \|  \| -   Pre-intervention \| 0/0 \|  \|  \|  \|  \| \|  \| -   Pulse oximetry \| 0/0 \|  \|  \|  \|  \| \|  \| -   Full O2 system \| 0/0 \| unable to compute \| unable to compute \| unable to compute \| unable to compute \|   Data are n/N (%) unless otherwise indicated. *Denominators vary according to the population included. ǂ Primary analysis compares Full Oxygen system and Pulse Oximetry periods. § Extended analysis compares Pulse Oximetry and Full Oxygen system periods to the Pre-intervention period. α Odds ratios are derived from multiple logistic regression models that are similar to those in the primary analysis, but do not adjust for clustering or time. γ Child under 15 years of age, excluding neonates. ALRI = cough or difficult breathing and any of: fast breathing, lower chest wall indrawing. CI = confidence interval. LBW = low birth weight, <2500 grams. Preterm defined as <37 weeks gestational age. |
| --- | --- | --- | --- | --- | --- | --- | --- | --- | --- | --- | --- | --- | --- | --- | --- | --- | --- | --- | --- | --- | --- | --- | --- | --- | --- | --- | --- | --- | --- | --- | --- | --- | --- | --- | --- | --- | --- | --- | --- | --- | --- | --- | --- | --- | --- | --- | --- | --- | --- | --- | --- | --- | --- | --- | --- | --- | --- | --- | --- | --- | --- | --- | --- | --- | --- | --- | --- | --- | --- | --- | --- | --- | --- | --- | --- | --- | --- | --- | --- | --- | --- | --- | --- | --- | --- | --- | --- | --- | --- | --- | --- | --- | --- | --- | --- | --- | --- | --- | --- | --- | --- | --- | --- | --- | --- | --- | --- | --- | --- | --- | --- | --- | --- | --- | --- | --- | --- | --- | --- | --- | --- | --- | --- | --- | --- | --- | --- | --- | --- | --- | --- | --- | --- | --- | --- | --- | --- | --- | --- | --- | --- | --- | --- | --- | --- | --- | --- | --- | --- | --- | --- | --- | --- | --- | --- | --- | --- | --- | --- | --- | --- | --- | --- | --- | --- | --- | --- | --- | --- | --- | --- | --- | --- | --- | --- | --- | --- | --- | --- | --- | --- | --- | --- | --- | --- | --- | --- | --- | --- | --- | --- | --- | --- | --- | --- | --- | --- | --- | --- | --- | --- | --- | --- | --- | --- | --- | --- | --- | --- | --- | --- | --- | --- | --- | --- | --- | --- | --- | --- | --- | --- | --- | --- | --- | --- | --- | --- | --- | --- | --- | --- | --- | --- | --- | --- | --- | --- | --- | --- | --- | --- | --- | --- | --- | --- | --- | --- | --- | --- | --- | --- | --- | --- | --- | --- | --- | --- | --- | --- | --- | --- | --- | --- | --- | --- | --- | --- | --- | --- | --- | --- | --- | --- | --- | --- | --- | --- | --- | --- | --- | --- | --- | --- | --- | --- | --- | --- | --- | --- | --- | --- | --- | --- | --- | --- | --- | --- | --- | --- | --- | --- | --- | --- | --- | --- | --- | --- | --- | --- | --- | --- | --- | --- | --- | --- | --- | --- | --- | --- | --- | --- | --- | --- | --- | --- | --- | --- | --- | --- | --- | --- | --- | --- | --- | --- | --- | --- | --- | --- | --- | --- | --- | --- | --- | --- | --- | --- | --- | --- | --- | --- | --- | --- | --- | --- | --- | --- | --- | --- | --- | --- | --- | --- | --- | --- | --- | --- | --- | --- | --- | --- | --- | --- | --- | --- | --- | --- | --- | --- | --- | --- | --- | --- | --- | --- | --- | --- | --- | --- | --- | --- | --- | --- | --- | --- | --- | --- | --- | --- | --- | --- | --- | --- | --- | --- | --- | --- | --- | --- | --- | --- | --- | --- | --- | --- | --- | --- | --- | --- | --- | --- | --- | --- | --- | --- | --- | --- | --- | --- | --- | --- | --- | --- | --- | --- | --- | --- | --- | --- | --- | --- | --- | --- | --- | --- | --- | --- | --- | --- | --- | --- | --- | --- | --- | --- | --- | --- | --- | --- | --- | --- | --- | --- | --- | --- | --- | --- | --- | --- | --- | --- | --- | --- | --- | --- | --- | --- | --- | --- | --- | --- | --- | --- | --- | --- | --- | --- | --- | --- | --- | --- | --- | --- | --- | --- | --- | --- | --- | --- | --- | --- | --- | --- | --- | --- | --- | --- | --- | --- | --- | --- | --- | --- | --- | --- | --- | --- | --- | --- | --- | --- | --- | --- | --- | --- | --- | --- | --- | --- | --- | --- | --- | --- | --- | --- | --- | --- | --- | --- | --- | --- | --- | --- | --- | --- | --- | --- | --- | --- | --- | --- | --- | --- | --- | --- | --- | --- | --- | --- | --- | --- | --- | --- | --- | --- | --- | --- | --- | --- | --- | --- | --- | --- | --- | --- | --- | --- | --- | --- | --- | --- | --- | --- | --- | --- | --- | --- | --- | --- | --- | --- | --- | --- | --- | --- | --- | --- | --- | --- | --- | --- | --- | --- | --- | --- | --- | --- | --- | --- | --- | --- | --- | --- | --- | --- | --- | --- | --- | --- | --- | --- | --- | --- | --- | --- | --- | --- | --- | --- | --- | --- | --- | --- | --- | --- | --- | --- | --- | --- | --- | --- | --- | --- | --- | --- | --- | --- | --- | --- | --- | --- | --- | --- | --- | --- | --- | --- | --- | --- | --- | --- | --- | --- | --- | --- | --- | --- | --- | --- | --- | --- | --- | --- | --- | --- | --- | --- | --- | --- | --- | --- | --- | --- | --- | --- | --- | --- | --- | --- | --- | --- | --- | --- | --- | --- | --- | --- | --- | --- | --- | --- | --- | --- | --- | --- | --- | --- | --- | --- | --- | --- | --- | --- | --- | --- | --- | --- | --- | --- | --- | --- | --- | --- | --- | --- | --- | --- | --- | --- | --- | --- | --- | --- | --- | --- | --- | --- | --- | --- | --- | --- | --- | --- | --- | --- | --- | --- | --- | --- | --- | --- | --- | --- | --- | --- | --- | --- | --- | --- | --- | --- | --- | --- | --- | --- | --- | --- | --- | --- | --- | --- | --- | --- | --- | --- | --- | --- | --- | --- | --- | --- | --- | --- | --- | --- | --- | --- | --- | --- | --- | --- | --- | --- | --- | --- | --- | --- | --- | --- | --- | --- | --- | --- | --- | --- | --- | --- | --- | --- | --- | --- | --- | --- | --- | --- | --- | --- | --- | --- | --- | --- | --- | --- | --- | --- | --- | --- | --- | --- | --- | --- | --- | --- | --- | --- | --- | --- | --- | --- | --- | --- | --- | --- | --- | --- | --- | --- | --- | --- | --- | --- | --- | --- | --- | --- | --- | --- | --- | --- | --- | --- | --- | --- | --- | --- | --- | --- | --- | --- | --- | --- | --- | --- | --- | --- | --- | --- | --- | --- | --- | --- | --- | --- | --- | --- | --- | --- | --- | --- | --- | --- | --- | --- | --- | --- | --- | --- | --- | --- | --- | --- | --- | --- | --- | --- | --- | --- | --- | --- | --- | --- | --- | --- | --- | --- | --- | --- | --- | --- | --- | --- | --- | --- | --- | --- | --- | --- | --- | --- | --- | --- | --- | --- | --- | --- | --- | --- | --- | --- | --- | --- | --- | --- | --- | --- | --- | --- | --- | --- | --- | --- | --- | --- | --- | --- | --- | --- | --- | --- | --- | --- | --- | --- | --- | --- | --- | --- | --- | --- | --- | --- | --- | --- | --- | --- | --- | --- | --- | --- | --- | --- | --- | --- | --- | --- | --- | --- | --- | --- | --- | --- | --- | --- | --- | --- | --- | --- | --- | --- | --- | --- | --- | --- | --- | --- | --- | --- | --- | --- | --- | --- | --- | --- | --- | --- | --- | --- | --- | --- | --- | --- | --- | --- | --- | --- | --- | --- | --- | --- | --- | --- | --- | --- | --- | --- | --- | --- | --- | --- | --- | --- | --- | --- | --- | --- | --- | --- | --- | --- | --- | --- | --- | --- | --- | --- | --- | --- | --- | --- | --- | --- | --- | --- | --- | --- | --- | --- | --- | --- | --- | --- | --- | --- | --- | --- | --- | --- | --- | --- | --- | --- | --- | --- | --- | --- | --- | --- | --- | --- | --- | --- | --- | --- | --- | --- | --- | --- | --- | --- | --- | --- | --- | --- | --- | --- | --- | --- | --- | --- | --- | --- | --- | --- | --- | --- | --- | --- | --- | --- | --- | --- | --- | --- | --- | --- | --- | --- | --- | --- | --- | --- | --- | --- | --- | --- | --- | --- | --- | --- | --- | --- | --- | --- | --- | --- | --- | --- | --- | --- | --- | --- | --- | --- | --- | --- | --- | --- | --- | --- | --- | --- | --- | --- | --- | --- | --- | --- | --- | --- | --- | --- | --- | --- | --- | --- | --- | --- | --- | --- | --- | --- | --- | --- | --- | --- | --- | --- | --- | --- | --- | --- | --- | --- | --- | --- | --- | --- | --- | --- | --- | --- | --- | --- | --- | --- | --- | --- | --- | --- | --- | --- | --- | --- | --- | --- | --- | --- | --- | --- | --- | --- | --- | --- | --- | --- | --- | --- | --- | --- | --- | --- | --- | --- | --- | --- | --- | --- | --- | --- | --- | --- | --- | --- | --- | --- | --- | --- | --- | --- | --- | --- | --- | --- | --- | --- | --- | --- | --- | --- | --- | --- | --- | --- | --- | --- | --- | --- | --- | --- | --- | --- | --- | --- | --- | --- | --- | --- | --- | --- | --- | --- | --- | --- | --- | --- | --- | --- | --- | --- | --- | --- | --- | --- | --- | --- | --- | --- | --- | --- | --- | --- | --- | --- | --- | --- | --- | --- | --- | --- | --- | --- | --- | --- | --- | --- | --- | --- | --- | --- | --- | --- | --- | --- | --- | --- | --- | --- | --- | --- | --- | --- | --- | --- | --- | --- | --- | --- | --- | --- | --- | --- | --- | --- | --- | --- | --- | --- | --- | --- | --- | --- | --- | --- | --- | --- | --- | --- | --- | --- | --- | --- | --- | --- | --- | --- | --- | --- | --- | --- | --- | --- | --- | --- | --- | --- | --- | --- | --- | --- | --- | --- | --- | --- | --- | --- | --- | --- | --- | --- | --- | --- | --- | --- | --- | --- | --- | --- | --- | --- | --- | --- | --- | --- | --- | --- | --- | --- | --- | --- | --- | --- | --- | --- | --- | --- | --- | --- | --- | --- | --- | --- | --- | --- | --- | --- | --- | --- | --- |
