# Supplementary material for: Oxygen systems to improve clinical care and outcomes for children and neonates: A stepped-wedge cluster-randomised trial in Nigeria
Source: PLoS Med. 2019 Nov 11;16(11):e1002951. doi: 10.1371/journal.pmed.1002951 (PMC6844455; doi:10.1371/journal.pmed.1002951)
Supplement: Attachment — Submitted filename: Author reply_20190807.docx [file pmed.1002951.s009.docx]

**Author reply to Editorial and Reviewer comments**

**"Oxygen systems to improve clinical care and outcomes for children and neonates: a stepped-wedge cluster-randomised trial." (PMEDICINE-D-19-02095)**

**We thank the Reviewers and Editors for their feedback. We really appreciate the amount of time and thought that went into the comments! Your suggestions have made this a more robust and readable paper. We hope that we have understood and answered all the questions satisfactorily, but would be glad to be contacted for any additional clarification.**

Dear Dr. Graham,

Thank you very much for submitting your manuscript "Oxygen systems to improve clinical care and outcomes for children and neonates: a stepped-wedge cluster-randomised trial." (PMEDICINE-D-19-02095) for consideration at PLOS Medicine.

Your paper was evaluated by a senior editor and discussed among all the editors here. It was also discussed with an academic editor with relevant expertise, and sent to independent reviewers, including a statistical reviewer. The reviews are appended at the bottom of this email and any accompanying reviewer attachments can be seen via the link below:

In light of these reviews, I am afraid that we will not be able to accept the manuscript for publication in the journal in its current form, but we would like to consider a revised version that addresses the reviewers' and editors' comments. Obviously we cannot make any decision about publication until we have seen the revised manuscript and your response, and we plan to seek re-review by one or more of the reviewers.

In revising the manuscript for further consideration, your revisions should address the specific points made by each reviewer and the editors. Please also check the guidelines for revised papers at <http://journals.plos.org/plosmedicine/s/revising-your-manuscript> for any that apply to your paper. In your rebuttal letter you should indicate your response to the reviewers' and editors' comments, the changes you have made in the manuscript, and include either an excerpt of the revised text or the location (eg: page and line number) where each change can be found. Please submit a clean version of the paper as the main article file; a version with changes marked should be uploaded as a marked up manuscript.

In addition, we request that you upload any figures associated with your paper as individual TIF or EPS files with 300dpi resolution at resubmission; please read our figure guidelines for more information on our requirements: <http://journals.plos.org/plosmedicine/s/figures>. While revising your submission, please upload your figure files to the PACE digital diagnostic tool, <https://pacev2.apexcovantage.com/>. PACE helps ensure that figures meet PLOS requirements. To use PACE, you must first register as a user. Then, login and navigate to the UPLOAD tab, where you will find detailed instructions on how to use the tool. If you encounter any issues or have any questions when using PACE, please email us at [PLOSMedicine@plos.org](mailto:PLOSMedicine@plos.org).

We expect to receive your revised manuscript by Aug 08 2019 11:59PM. Please email us ([plosmedicine@plos.org](mailto:plosmedicine@plos.org)) if you have any questions or concerns.

***Please note while forming your response, if your article is accepted, you may have the opportunity to make the peer review history publicly available. The record will include editor decision letters (with reviews) and your responses to reviewer comments. If eligible, we will contact you to opt in or out.***

We ask every co-author listed on the manuscript to fill in a contributing author statement, making sure to declare all competing interests. If any of the co-authors have not filled in the statement, we will remind them to do so when the paper is revised. If all statements are not completed in a timely fashion this could hold up the re-review process. If new competing interests are declared later in the revision process, this may also hold up the submission. Should there be a problem getting one of your co-authors to fill in a statement we will be in contact. YOU MUST NOT ADD OR REMOVE AUTHORS UNLESS YOU HAVE ALERTED THE EDITOR HANDLING THE MANUSCRIPT TO THE CHANGE AND THEY SPECIFICALLY HAVE AGREED TO IT. You can see our competing interests policy here: <http://journals.plos.org/plosmedicine/s/competing-interests>.

Please use the following link to submit the revised manuscript:

<https://www.editorialmanager.com/pmedicine/>

Your article can be found in the "Submissions Needing Revision" folder.

To enhance the reproducibility of your results, we recommend that you deposit your laboratory protocols in protocols.io, where a protocol can be assigned its own identifier (DOI) such that it can be cited independently in the future. For instructions see <http://journals.plos.org/plosmedicine/s/submission-guidelines#loc-methods>.

Please ensure that the paper adheres to the PLOS Data Availability Policy (see <http://journals.plos.org/plosmedicine/s/data-availability>), which requires that all data underlying the study's findings be provided in a repository or as Supporting Information. For data residing with a third party, authors are required to provide instructions with contact information for obtaining the data. PLOS journals do not allow statements supported by "data not shown" or "unpublished results." For such statements, authors must provide supporting data or cite public sources that include it.

We look forward to receiving your revised manuscript.

Sincerely,

Clare Stone, PhD

Managing Editor

PLOS Medicine

plosmedicine.org

-----------------------------------------------------------

**Requests from the editors:**

In the abstract and elsewhere, you report "no effect ... on death" for children with the oxygen system (CIs cross unity), and "may have increased ... neonatal odds of death" (CIs do not cross unity). So, it may appear as though you believe a negative result but are more sceptical about a positive result (of an undesirable outcome). Please address. I'd suggest adopting less equivocal language ... i.e. noting an observed increase in risk of death in the neonatal group, which you can then discuss possible reasons for.

**Thank you for your comment. We accept the need to be clear about statistical findings in reporting results. We have revised the language accordingly.**

**More broadly, we aimed to be circumspect in our interpretation of the data – for positive, negative, or neutral findings – given that our study had low power and we reported multiple clinical outcomes. We were particularly cautious regarding neonatal outcomes, as these were derived from a smaller population that mostly represented the 4 larger hospitals (in which there was particularly striking heterogeneity of outcomes). As such, we used language that reflected this uncertainty and aimed to interpreted effect estimates and confidence intervals in context.**

Please be more explicit about the limitations of the study in the abstract – starting the final sentence of the Methods and Findings section with ‘Limitations of our study are…’

**Revised as requested.**

**“Limitations of our study are: lower than anticipated power to detect change in mortality outcomes (low event rates, low participant numbers, high intra-cluster correlation); major contextual changes related to the 2016-17 Nigerian economic recession that influenced care-seeking and hospital function during the study period, potentially confounding mortality outcomes.”**

Please provide p values with Cis in the abstract

**We have include p values and Cis in the abstract, and added p values to the results.**

Data – can you please clarify is all users can access data at osf.io/pumdq and can you please provide a direct link to the data set in this location if it’s a large depository?

**Yes, this is Public. This repository does not have direct links, but has an easy to use search function through which this dataset can be found. The official citation is copied below, and included in the manuscript.**

Graham H. Oxygen systems to improve clinical care and outcomes for children and neonates: a stepped-wedge cluster-randomised trial [Internet]. OSF; 23 Jul 2019. Available: osf.io/pumdq

At this stage, we ask that you include a short, non-technical Author Summary of your research to make findings accessible to a wide audience that includes both scientists and non-scientists. The Author Summary should immediately follow the Abstract in your revised manuscript. This text is subject to editorial change and should be distinct from the scientific abstract. Please see our author guidelines for more information: <https://journals.plos.org/plosmedicine/s/revising-your-manuscript#loc-author-summary>

**Why Was This Study Done?**

- Oxygen therapy is important for many acute medical conditions, particularly among unwell children and newborns in whom hypoxaemia (low blood oxygen) is common.
- Oxygen access and use is suboptimal in many hospitals in low- and middle-income countries.
- Improved oxygen systems may reduce deaths from pneumonia.
- To scale up oxygen in resource-limited settings, we need better information about how to improve oxygen systems, and stronger evidence on the benefits for newborns and children.

**What Did the Researchers Do and Find?**

- We introduced pulse oximetry and improved oxygen systems in 12 Nigerian hospitals, aiming to provide continuous oxygen therapy for every child and neonate who needed it.
- We evaluated the impact of pulse oximetry and improved oxygen systems on care practices and clinical outcomes for >24,000 unwell newborns and children.
- We found that the improved oxygen system had no effect on outcomes for children, when compared against the introduction of pulse oximetry. However, pulse oximetry may have reduced the risk of death from pneumonia by approximately 50% compared to baseline.
- We found that the improved oxygen system was associated with a 45% increase in neonatal death, when compared against the introduction of pulse oximetry. However, neither pulse oximetry nor the full oxygen system had any effect on neonatal death when compared against baseline.

**What Do These Findings Mean?**

- Pulse oximetry should be central to all activities aiming to improve access to oxygen therapy.
- Where some oxygen is already available, the introduction of pulse oximetry may improve how oxygen is used and may reduce deaths from pneumonia. However, the added benefit of further improvements to oxygen systems may be minimal.
- The negative results for newborns is surprising, but should be interpreted cautiously. Analysis of results from individual hospitals shows significant variability in outcomes for newborns and children.
- Our study was challenged by a major economic recession, resulting in unexpected changes in admission numbers, illness severity, and care practices, and low power to detect change in clinical outcomes.

Please use square brackets for refs in the main text.

**Updated to PLoS formatting for references.**

Did your study have a prospective protocol or analysis plan? Please state this (either way) early in the Methods section.

a) If a prospective analysis plan (from your funding proposal, IRB or other ethics committee submission, study protocol, or other planning document written before analyzing the data) was used in designing the study, please include the relevant prospectively written document with your revised manuscript as a Supporting Information file to be published alongside your study, and cite it in the Methods section. A legend for this file should be included at the end of your manuscript.

b) If no such document exists, please make sure that the Methods section transparently describes when analyses were planned, and when/why any data-driven changes to analyses took place.

c) In either case, changes in the analysis—including those made in response to peer review comments—should be identified as such in the Methods section of the paper, with rationale.

**We had a prospective protocol that was published and is referenced early in the Methods section. We had a prospective analysis plan specifically for the mixed-effects model, recorded alongside notes on the discussion and decision making in our research log book (but not available in a specific document). We have added a reference to “pre-specified analysis plan” in the Methods section (analytical models). There were no deviations from this analysis plan (apart from adding p values on the request of the Editor and Statistical Reviewer and conducting post-hoc analysis on the hypoxaemia and <5yo subgroups on the request of Reviewer 3 – results provided in web-Appendix).**

Please provide a flow chart of the trial participants, arms and exclusions etc, per standard for trial manuscripts. Apologies if I am missing this.

**We have added this as a supplemental file.**

Please provide a CONSORT reporting checklist – as a supp file.

**We have added the CONSORT 2010 Extension for Cluster Trials as a supplemental file.**

**Comments from the reviewers:**

Reviewer #1: The authors are to be congratulated on addressing a very important bur relatively neglected topic, namely how best to use oxygen in the care of sick children in low or middle income countries. The results of the trial are rather surprising, especially the possible increase in neonatal mortality in the oxygen intervention group, which may have been due to chance, but could be true in which case this is an important and rather alarming finding which needs further investigation.

Major points

The trial has a number of weakness which are largely covered in the discussion, such as the fact that it was under-powered to detect a small difference between groups, but I think that there are a couple of additional ones that could be mentioned in the discussion.

a. The trial design. A stepped wedge design was chosen rather than a contemporary, randomised study for sound logistic reasons but step wedged trials only work well when risk and potential confounders remain relatively stable throughout the period of the study. For reasons outside the control of the investigators, this was not the case in this study, and this could have influenced the outcome. An attempt to take this into consideration has been made through use of a severity score but this is still a concern.

**Thank you. We have revised the limitations section to make this study design limitation clearer.**

**“Using a stepped-wedge design enabled us to gain the benefits of randomisation and learn valuable lessons about implementation along the way, but it did make adjusting for temporal effects harder than if it were a parallel cluster trial. Stepped-wedge designs work best when potential confounders remain relatively stable during the study period, and this may have been compromised by the major economic and social changes in Nigeria that likely introduced additional confounding.”**

b. Heterogeneity between hospitals. Facilities and numbers of trained staff varied widely between hospitals, for example between hospitals H1 and H9. An attempt was made to overcome this problem in matching the intervention groups through stratification but this does not take account of the fact that the oxygen intervention might have had variable impacts in hospitals with well or poorly developed oxygen supplies, infrastructure and staff. An attempt to look at this issue is given by the presentation of results from each hospital but numbers were too small to undertake a definitive sub-group analysis. This issue could be mentioned in the discussion.

**Thank you. We agree that the observed heterogeneity suggests that the multi-faceted intervention may have worked differently in different contexts, and that small numbers of participants in individual hospitals (and the lack of a good counterfactual) makes subgroup analysis challenging. We have revised this section of the Discussion to explicitly acknowledge this.**

**“Analysis of outcomes on a hospital-by-hospital basis shows significant heterogeneity (Web-appendix 5) and it is possible that the multi-faceted intervention worked differently in different contexts. For example, H1 was a large mission hospital that maintained services relatively unchanged throughout the economic recession and realised mortality reductions for both children and neonates. In contrast, H7 was a large government hospital that provided free care to patients and recorded few deaths pre-intervention but was strongly impacted by the economic recession (including introducing patient fees and cutting services). H7 realised mortality increases for both children and neonates. Small study numbers in individual hospitals makes definitive subgroup analysis challenging. However preliminary exploration of this heterogeneity has identified a number of possible contextual and mechanistic factors that will be evaluated more fully in a separate paper.”**

Other points

a. Pre-intervention data. Pre-intervention data were collected retrospectively whilst in the intervention periods they were collected prospectively using a standard CRF. Data from the pre-intervention period are likely to have been much less complete, and perhaps less reliable, than that that collected prospectively. How was this issue handled?

**Thank you. We have revised the text to make this clear. You are correct in guessing that the retrospectively-collected pre-intervention data was less complete. However, we were fortunate to have very low rates of missing outcome data throughout all periods (1.3% in retrospective period, 0.3% in prospective period). Our pre-specified analysis plan was to exclude participants with missing data from analysis. In the context of expecting more missing data in the pre-intervention period, this was considered to be the conservative approach (i.e. bias towards less benefit). We considered re-analysing using different approaches to dealing with missing data, but our statisticians advised that this would not be worthwhile given such low rates of missing data.**

**METHODS: “We excluded participants with missing data from analysis.”**

**DISCUSSION: “We had complete clinical outcome data for 24778/24859 (99.67%) of participants in the pulse oximetry and full oxygen system periods (prospective data collection), and 23812/24117 (98.74%) of participants in the pre-intervention period (retrospective data collection).”**

b. Impact on clinical performance. Introduction of the oxygen system was accompanied by training (appendix 2)) but it is not clear how focused this was and whether there was some additional, more general training provided on management of the sick child which could have influenced outcome in addition to the input of oxygen therapy.

**The training was focussed on pulse oximetry and oxygen therapy. We aimed to integrate it within existing WHO guidelines (Pocket book of hospital care for children; Oxygen therapy for children), and we provided hospitals with hard copies of these guidelines. We did not do training more broadly. It is possible that hospitals might organise additional training, or be inspired to address broad quality of care issues, and we are currently exploring this in our realist process evaluation.**

**We have revised the web-Appendix 2 to be more explicit about the focus on pulse oximetry and oxygen therapy.**

c. Role of initial hypoxaemia. Was a sub-analysis done of impact on children who were hypoxaemic at the initial assessment or were the guidelines on when to give oxygen followed so assiduously that it was not possible to do this?

**Thank you. Reviewer 3 also asked about impact on the hypoxaemic cohort and why we did not use them as the primary population of interest, so we will address this in some detail.**

**The short answer is that, yes, we have analysed the primary clinical outcomes restricted to the hypoxaemic cohort and this is now included as Web-appendix 6. These results are qualitatively similar to the main results table, but with lesser precision.**

**However, we urge particular caution interpreting these for a number of reasons. First, pulse oximetry was rarely conducted during the pre-intervention period (a single hospital) so we cannot make any meaningful comparisons against the pre-intervention period. Second, pulse oximetry improved over the first few months of the pulse oximetry period. As such the ‘hypoxaemic’ population in each epoch may not genuinely be comparable, especially in a stepped wedge design where different hospitals contribute different amounts of data to different study periods. Indeed, this may contribute to additional confounding (i.e. documentation of a SpO_2_ result may be associated with both the outcome of interest and the study period).**

**A more consistent measure of hypoxaemia is using documented clinical signs of hypoxaemia, so Web-appendix 6 also includes analysis of those who had any sign of hypoxaemia on admission (e.g. very fast breathing, severe respiratory distress, central cyanosis), ignoring the SpO_2_ result. This allowed for comparison across all time periods and probably gave us more comparable populations (although there may still have been different documentation practices between hospitals). The influence of this hypoxaemic population should also have been captured in our ‘severity-adjusted’ analysis (since the major signs of hypoxaemia were also signs of illness severity that were reflected in our analytical model).**

**We did not use hypoxaemic patients as the primary population for two reasons. First, this was operational research in 12 heterogeneous hospitals so we wanted a broad and generic inclusion criteria. Hence, we included all admitted children (<15 years) and neonates (<28 days), and the diagnostic subgroups that we thought would realise greatest benefit from an improved oxygen system (pneumonia and preterm/LBW neonates) as our primary outcome populations. Second, we did not have baseline data on hypoxaemia and did not know how well pulse oximetry practices would be adopted by different hospitals. Previous studies in Papua New Guinea and Laos had reached 50-80% pulse oximetry coverage on the target population, which we did not think would adequately provide comparable populations across study periods or between hospitals.**

d. Sustainability. Do the investigators have any information on what has happened in the trial hospitals since the study finished? Has there been any long-term impact or has the situation reverted to pre-intervention days?

**We are planning medium-term follow-up in 2020. Anecdotal reports suggest that the equipment is continuing to be well used, and is in good working condition. Our team gets occasional requests for assistance with repairs, or with procuring new equipment (for scaling up to other parts of the hospital).**

e. Messages. A message can be disseminated to paediatricians working in LMIC that results from this study support the findings from a limited number of previous studies (Laos, PNG) that giving oxygen to children with severe pneumonia reduces mortality. However, it is not clear what the message about neonates should be. Should they not be given oxygen? How could this issue be clarified? Some guidance from investigators who have made a major investment in this area of paediatric research and care would be helpful.

**Thank you. We have had a lot of discussion about how to responsibly communicate our findings. We have revised some of the Discussion and added a paragraph to the Practical implications section:**

**“Oxygen, administered as low-flow oxygen or with continuous positive airway pressure (CPAP), is an important part of neonatal care. We advise readers against making changes to neonatal oxygen policies or practices on the basis of this study, and to adhere to WHO guidelines[32]. Oxygen, like any medication, should be given judiciously, accompanied by treatment of the specific medical condition and good supportive care. Future research and quality improvement activities should seek to better understand how oxygen can be used most safely and effectively neonatal care – particularly for the most at-risk preterm neonates.”**

Reviewer #2: Statistical review

This paper reports a stepped wedge cluster randomised trial evaluating the rolling out of an intervention for improving hospital oxygen systems for treatment children with acute lower respiratory infection. The rolling out was in two phases: pulse oximetry and then multi-faceted oxygen system. The primary analysis is full vs pulse, although both are compared to baseline also.

The trial had a number of practical issues occur beyond the control of the authors. This may have caused more than usual confounding. I think the authors do an excellent job of discussing this and the potential implications in light of the unexpected results from the trial. Generally the statistical methods and reporting is also very good quality. I have some comments, listed below:

1.	Line 51 (and line 289) 'no effect on odds' - 'no significant association' or 'no evidence of effect on odds'. Since this was a trial testing a specific hypothesis, I recommend p-values are provided in addition to CIs.

**We have added p values to the abstract and the main results tables.**

2.	Lines 54-56: provide effect size and CI.

**Effect sizes and CIs added.**

3.	Abstract and results: personally I think p-values can add useful information when reported in addition to CIs: this is clearly a prospectively planned study testing pre-specified hypotheses rather than an exploratory one where p-values might be inappropriate. However the published protocol mentions just effect sizes and CIs would be reported so the lack of p-values is consistent with what was planned.

**We have added p values to the abstract and the main results tables.**

4.	Line 57: "Our study had lower than anticipated power to detect change in mortality outcomes." - briefly say why? Lower event rate than anticipated? Unclear whether the next sentence is the explanation but if a link between this and the reason for lower power could be given, this would be clearer.

**Thank you. We have added a brief explainer that is consistent with the more detailed explanation provided in the main text.**

**“Limitations of our study are: lower than anticipated power to detect change in mortality outcomes (low event rates, low participant numbers, high intra-cluster correlation); major contextual changes related to the 2016-17 Nigerian economic recession that influenced care-seeking and hospital function during the study period, potentially confounding mortality outcomes.”**

5.	Intervention section - can more be said in this section about the pulse oximetry component and how much earlier that was available compared to the full intervention?

**Thank you. We have revised for clarity.**

**“We commenced prospective data collection and introduced pulse oximetry to all hospitals in November 2015. We introduced pulse oximetry prior to implementation of the full oxygen system to enable quantification of hypoxaemia prevalence and evaluation of the effect of the introduction of pulse oximetry alone (extended analysis). Hospitals received the full oxygen system in March 2016, July 2016, November 2016, or March 2017, in the randomised order. We conducted retrospective data collection in late 2015, covering the pre-intervention period (November 2013 to October 2015) for extended analysis.”**

6.	I was a bit confused by the use of 'mortality' as the primary outcome and then 'pneumonia CFR' in the power calculation. Are these are completely the same? I'd have thought that all-cause mortality and pneumonia CFR might not be completely identical.

**We have revised to include the power calculations for the four primary clinical outcomes (mortality in children overall, children with LRTI, neonates overall, and preterm/LBW neonates).**

**“Our original estimation contained a mathematical error, and after correction we calculated approximately 50% power to detect a 20% reduction in overall child mortality (4% to 3.2%, ICC 0.018), 35% power to detect a 35% reduction in child pneumonia mortality (6.1% to 4.0%, ICC 0.032), 50% power to detect a 20% reduction in neonatal mortality (8.2% to 6.6%, ICC 0.063), and 55% power to detect a 25% reduction in preterm/LBW mortality (18% to 13.5%, ICC 0.123).”**

7.	Sample size calculation: can the assumed rate of pneumonia CFR in the pulse oximetry and full intervention period be provided?

**Revised. See above.**

8.	Line 226-227: can a bit more detail about the random hospital-time interaction effects be added? It was not clear how this was done.

**We included a variance component for random hospital (cluster) by time (4-month step) effects in the analysis model, and compared this model with the simpler model that assumed random hospital effects only (constant over time), using a likelihood ratio test.**

**“Our primary analysis model included fixed effects for intervention and time (4-month steps), with random effects for cluster (hospital). To decide whether extensions to the basic model were warranted we looked for evidence of hospital-time interaction (using a variance component for random by time effects in an extended analysis model, and comparing this with the simpler model using a likelihood ratio test)[45]. We found no evidence of hospital-time interaction for the primary clinical outcome (death). However, there was evidence of interaction for the practice outcomes and we adjusted models accordingly.”**

**Hemming K, Taljaard M, Forbes A. Analysis of cluster randomised stepped wedge trials with repeated cross-sectional samples. Trials. 2017;18(1):101. doi: 10.1186/s13063-017-1833-7. PubMed PMID: 28259174; PubMed Central PMCID: PMCPMC5336660.**

9.	Line 291-292: although I agree the broad conclusions are the same the severity adjusted model seems to have substantially strong evidence of an increase in odds for neonates.

**Correct. We have added a note to this effect.**

10.	Is table 3 needed when it seems like all the information is included in table 4?

**Thank you. Previous internal review suggested that we should present the Primary Outcome results in a separate table for easier viewing. But you are correct, all the information from table 3 is included in table 4. We are happy to defer to the Editor’s preference.**

James Wason

Reviewer #3: General comments

This is a peer review of the manuscript entitled "Oxygen systems to improve clinical care and outcomes for children and neonates: a stepped-wedged cluster randomised trial" by Graham and colleagues. This report is of results from a stepped-wedge cluster randomized trial evaluating the effects of oxygen system introduction in 12 Nigerian hospitals on mortality and clinical practices.

This is an extremely important topic and the question addressed is also extremely important. I want to first off acknowledge the major efforts that were undertaken to conduct this trial and the incredible challenges that were faced to execute this work. Hats off. For me there are 5 main observations after reading and reflecting on this important work.

1)	Due to an unfortunate miscalculation the study was substantially underpowered for its main outcomes of oxygen systems implementation on hospital mortality (35-50% power for the effect sizes listed). Thus the main findings of no difference in mortality among children between the pulse oximetry and oxygen system time periods is not surprising. It is notable though that the authors found a surprising increased odds of mortality among neonates, but they have also acknowledged that given the lack of power this finding also may be due to chance and should be interpreted with caution. This neonatal outcome is potentially very important and deserves attention.

**Thank you. We agree with the need to be cautious about interpreting results, while taking potentially significant positive and negative findings seriously. We hope that we have achieved this balance appropriately.**

2)	Due to unfortunate instability in Nigeria there were major health system disruptions that could not really be accounted for in the analyses. It appears that roughly half of the hospitals were in the pulse oximetry period during this instability (and this period has 4,000+ less observations than the oxygen system period), which may have further compromised this period and falsely lowered mortality during this period (thus confounding results). This raises further uncertainty to any findings and additionally complicates having limited power to start with (likely further lowered power also).

**Correct.**

3)	Secondary exploratory outcomes showed lower odds of death for ALRI only among children in the pulse ox and oxygen system periods, compared to the pre-intervention period; but misclassification could be an issue in the pre-intervention period since this data was collected differently (retrospective) (the authors state in the limitations section that misclassification shouldn't be an issue based on audits)? I would encourage the authors to include the audit data in their report to support this as it would strengthen their findings that the pre-intervention period is valid and comparable to the intervention period.

**See detailed answer below.**

4)	Some key results, and the ensuing interpretation, seem to counter one another, or are at least not completely supportive (or maybe I'm not understanding them fully)? Specifically, secondary outcome analyses showed that compared to the pre-intervention period, the pulse oximetry period and the full oxygen period both reduced the odds of death for children with ALRI. But the practice outcomes analysis shows that while pulse oximetry practices improved dramatically, the pulse oximetry period DID NOT have any impact on overall oxygen use or perhaps even more importantly DID NOT have any impact on oxygen use for those with hypoxemia. I'm having a hard time fitting this together into a coherent story given the children most likely to benefit would presumably be those that are hypoxemic.

On the other hand, the practice outcomes also show that oxygen practices improved (but to a lesser extent than pulse oximetry practices) when the full oxygen system was introduced, and this could make sense. But there must be something else happening to explain why pulse ox alone reduced mortality when it didn't change oxygen use, especially among those most in need of it? I could not find any conclusive explanation in the data presented, but I fully understand the challenges faced during this study.

**Heatmap data shows that most hospitals increased their oxygen coverage to >80% of genuinely hypoxaemic children within 3-6 months of pulse oximetry introduction (from <<50%) – without substantial changes in overall oxygen use. This substantial change in practice is reflected in the adjusted effect estimates to a lesser extent, within the limitations of practice definitions and analysis models (the mixed-effects model does adjust for variable intervention effect within a study period).**

**For children, oxygen use overall did not change during the pulse oximetry period (aOR 0.72; 0.43-1.19) compared to the pre-intervention period, but it did increase in the full oxygen system period (aOR 1.50; 1.04-2.15). There was a suggestion of better use for those with SpO_2_<90% in both the pulse oximetry period (aOR 2.85; 0.19-43.63) and the full oxygen system period (aOR 6.90; 0.52-91.70) but with gross imprecision due to limited pre-intervention SpO2 data. In contrast, the proportion of children with signs of hypoxaemia receiving oxygen therapy did not change in the pulse oximetry period (aOR 1.00; 0.51-1.95), but increased in the full oxygen system period (aOR 1.71; 1.06-2.75). However, ‘signs of hypoxaemia’ is a much looser definition of hypoxaemia than using SpO_2_ - so this finding is still consistent with better targeting of oxygen therapy for truly hypoxaemic children (SpO_2_<90%).**

**The “indication for oxygen” outcome attempts to reflect targeting of oxygen therapy (i.e. did patients receiving oxygen have a valid reason for it?). This suggests a trend towards more targeted use in the pulse oximetry period (aOR 1.21; 0.55-2.68) and more liberal use in the full oxygen system period (aOR 0.73; 0.41-1.31) – but with imprecision. However, this uses a composite definition of hypoxaemia, consistent with our clinical guideline – i.e. “administer oxygen on the basis of SpO_2_, but if SpO_2_ available use clinical signs”.**

**Indication for oxygen = # with SpO_2_<90% on admission (or signs of hypoxaemia if no SpO_2_ documented) / # prescribed oxygen therapy.**

**The marked change in pulse oximetry use over the study periods clearly confounds these results. The alternative is to use a different definition of hypoxaemia:**

1. **SpO_2_<90% if got oxygen = # with SpO_2_<90% on admission / # prescribed oxygen therapy**
2. **Hypox signs if got oxygen = # with signs of hypoxaemia / # prescribed oxygen therapy**

**(For the reviewer’s benefit we have included results obtained from the two alternative definitions in the table below.) Each of these approaches have biases, but the crude trend in each suggests more targeted oxygen use for children in the pulse oximetry period.**

**Using the SpO2 definition gets closer to what is “appropriate” when pulse oximetry is routinely used, but it makes comparisons to the pre-intervention period difficult (very little pulse oximeter use) and means the pulse oximetry and full oxygen system period numerators are not truly comparable (15% of patients in the pulse oximetry period did not have an SpO_2_ recorded).**

**Using the clinical signs definition for hypoxaemia may allow for more meaningful comparison across periods (assuming pulse oximetry use did not change documentation of clinical signs), but substantially underestimates the “appropriate” use during the pulse oximetry and full oxygen system periods (in which healthcare workers were appropriately not giving oxygen to patients with clinical signs but SpO_2_>90%).**

**Our use of the composite measure was pre-specified, intended to give the most balanced indication of appropriate oxygen targeting and consistent with the clinical guidelines. But we acknowledge that is also influenced by the aforementioned bias and underestimates the “appropriate” use during the pulse oximetry and full oxygen system periods.**

**We have added a note to the results table and the Results section to better explain this and be true to the results.**

|  | **Proportions** | **Mixed-model adjusted odds ratio (95% CI)** α | | | | **ICC**β |
| --- | --- | --- | --- | --- | --- | --- |
|  | **n/N (%)*** | **Primary analysisǂ** | | **Extended analysis§** | | **(95%CI)** |
| ***Neonates*** |  |  | **p value** |  | **p value** |  |
| Indication for oxygen   - Pre-intervention - Pulse oximetry - Full O_2_ system | 1002/1794 (55.9%)  365/559 (65.3%)  858/1416 (60.6%) | -  1.42 (0.77-2.63) | 0.265 | -  2.00 (0.80-4.95)  2.09 (1.09-4.02) | 0.136  0.027 | 0.07  (0.02-0.19) |
| SpO_2_<90% if got oxygen   - Pre-intervention - Pulse oximetry - Full O_2_ system | 24/1794 (1.3%)  307/559 (54.9%)  833/1416 (58.8%) | -  1.59 (0.89-2.84) | 0.120 | -  62.34 (0.13.66-284.59)  110.54 (33.19-368.19) | <0.001  <0.001 | 0.11  (0.03-0.30) |
| Hypox signs if got oxygen   - Pre-intervention - Pulse oximetry - Full O_2_ system | 1036/1794 (57.8%)  345/556 (61.7%)  693/1416 (48.9%) | -  0.66 (0.46-0.94) | 0.022 | -  1.39 (0.81-2.39)  0.73 (0.49-1.08) | 0.230  0.113 | 0.03  (0.01-0.10) |
| ***Children*** |  |  |  |  |  |  |
| Indication for oxygen   - Pre-intervention - Pulse oximetry - Full O_2_ system | 778/1184 (65.7%)  311/455 (68.4%)  727/1262 (57.6%) | -  0.83 (0.51-1.35) | 0.457 | -  1.21 (0.55-2.68)  0.73 (0.41-1.31) | 0.634  0.291 | 0.03  (0.01-0.11) |
| SpO_2_<90% if got oxygen   - Pre-intervention - Pulse oximetry - Full O_2_ system | 34/1184 (2.8%)  265/455 (65.3%)  694/1262 (55.0%) | -  1.07 (0.67-1.70) | 0.775 | -  44.90 (11.30-178.45)  44.15 (13.60-143.26) | <0.001  <0.001 | 0.07  (0.02-0.19) |
| Hypox signs if got oxygen   - Pre-intervention - Pulse oximetry - Full O_2_ system | 810/1184 (68.4%)  327/455 (71.9%)  755/1262 (59.8%) | -  0.79 (0.51-1.21) | 0.272 | -  0.94 (0.50-1.77)  0.66 (0.41-1.05) | 0.845  0.080 | 0.10  (0.04-0.23) |

5)	I would like the authors to include much more analyses on the outcomes of children and neonates with hypoxemia. I find this critical subgroup missing throughout and given the population most likely to benefit from the intervention are hypoxemic children and neonates this should be presented with the main results. (in my view these children and neonates should have been the target population for the primary outcome). This data should be available given the pulse oximetry period involved pulse oximetry introduction and this data should be included with this report. Please also include data on the quality of pulse oximetry measurements. Please also clarify why older children aged 5-14 or even aged 2-5 years were included in the primary analysis given most mortality is experienced among children less than 2 years of age? Inclusion of this older population with less mortality may have further diluted the mortality effect of the oxygen system possibly but this is not discussed anywhere.

**Thank you. Reviewer 1 also asked about impact on the hypoxaemic cohort.**

**The short answer is that, yes, we have analysed the primary clinical outcomes restricted to the hypoxaemic cohort and this is now included as Web-appendix 6. These results are qualitatively similar to the main results table, but with lesser precision.**

**However, we urge particular caution interpreting these for a number of reasons. First, pulse oximetry was rarely conducted during the pre-intervention period (a single hospital) so we cannot make any meaningful comparisons against the pre-intervention period. Second, pulse oximetry improved over the first few months of the pulse oximetry period. As such the ‘hypoxaemic’ population in each epoch may not genuinely be comparable, especially in a stepped wedge design where different hospitals contribute different amounts of data to different study periods. Indeed, this may contribute to additional confounding (i.e. documentation of a SpO_2_ result associated with both the outcome of interest and the study period).**

**A more consistent measure of hypoxaemia is using documented clinical signs of hypoxaemia, so Web-appendix 6 also includes analysis of those who had any sign of hypoxaemia on admission (e.g. very fast breathing, severe respiratory distress, central cyanosis), ignoring the SpO_2_ result. This allowed for comparison across all time periods and probably gave us more comparable populations (although there may still have been different documentation practices between hospitals). The influence of this hypoxaemic population should also have been captured in our ‘severity-adjusted’ analysis (since the major signs of hypoxaemia were also signs of illness severity that were reflected in our analytical model).**

**We did not use hypoxaemic patients as the primary population for two reasons. First, this was operational research in 12 heterogeneous hospitals so we wanted a broad and generic inclusion criteria. Hence, we included all admitted children (<15 years) and neonates (<28 days), and the diagnostic subgroups that we thought would realise greatest benefit from an improved oxygen system (pneumonia and preterm/LBW neonates) as our primary outcome populations. Second, we did not have baseline data on hypoxaemia and did not know how well pulse oximetry practices would be adopted by different hospitals. Previous studies in Papua New Guinea and Laos had reached 50-80% pulse oximetry coverage on the target population, which we did not think would adequately provide comparable populations across study periods or between hospitals.**

**Regarding the age choice, the reviewer makes a fair observation: the majority of deaths are in younger children in whom the expected benefit may have been greater. Previous studies have focused on children under the age of five years. To advance the science we were eager to include neonates and older children. Children under the age of five years constituted ~75% of the child cohort, but it is possible that the 25% of older children may have diluted the intervention effects. Please note, the severity-adjusted analysis include adjustment for age.**

| Neonate (≤ 28 days) | 8813 (36.5) | 2983 (29.1) | 4733 (32.4) |
| --- | --- | --- | --- |
| Infant (1-12 months) | 4709 (19.5) | 2218 (21.6) | 2520 (17.3) |
| Young child (1-5 years) | 7788 (32.3) | 3580 (34.9) | 4803 (32.9) |
| Older child (5-15 years) | 2559 (10.6) | 1452 (14.1) | 2458 (16.8) |
| Child (age unknown) | 248 (1.0) | 34 (0.3) | 78 (0.5) |
| Age, months median (IQR) | 9.0 (0.1-24.0) | 11.1 (0.3-31.0) | 12.0 (0.1-36.0) |
| Age, months mean (SD) | 21.0 (32.2) | 24.8 (35.7) | 27.0 (37.2) |

**We have included subgroup analysis of the primary clinical outcomes focussed on children under the age of five years in Web-appendix 6. Table copied below. The findings are qualitatively similar to the primary outcome results.**

|  |  | **Mixed-model adjusted odds ratio (95% CI)** α | | | | | | | |  |
| --- | --- | --- | --- | --- | --- | --- | --- | --- | --- | --- |
|  | **Deaths** | **Primary analysisǂ** | | | | **Extended analysis§** | | | | **ICC**β |
|  | **n/N (%)*** | **Basic model** | | **severity-adjusted** | | **Basic model** | | **severity-adjusted** | | **(95%CI)** |
| **A – Children under 5 years of age (excluding neonates)** | |  | **P** |  | **P** |  | **P** |  | **P** |  |
| Child   - Pre-intervention - Pulse oximetry - Full O_2_ system | 511/12559 (4.1)  187/5145 (3.6)  342/6883 (5.0) | -  1.08 (0.75-1.54) | 0.691 | -  1.16 (0.78-1.74) | 0.457 | -  1.25 (0.78-2.01)  1.16 (0.83-1.63) | 0.352  0.383 | -  1.12 (0.65-1.94)  1.18 (0.80-1.76) | 0.673  0.406 | 0.05  (0.02-0.10) |
| Child ALRI   - Pre-intervention - Pulse oximetry - Full O_2_ system | 108/1786 (6.1)  41/689 (6.0)  87/1072 (8.1) | -  1.00 (0.43-2.29) | 0.997 | -  1.32 (0.54-3.20) | 0.544 | -  0.45 (0.17-1.22)  0.51 (0.27-0.95) | 0.117  0.033 | -  0.33 (0.11-0.99)  0.46 (0.23-0.92) | 0.133  0.027 | 0.10  (0.04-0.23) |

*Denominators vary according to the population included. ǂ Primary analysis compares Full Oxygen system and Pulse Oximetry periods. § Extended analysis compares Pulse Oximetry and Full Oxygen system periods to the Pre-intervention period. α Mixed-model odds ratios account for the clustering of patients within hospitals and adjust for time trends. Under the stepped-wedge design, the adjusted odds ratios are calculated with the use of all data points in the intervention period versus the comparison period and therefore represent the average odds of exposure to the intervention. β Intra-cluster correlation coefficient (ICC).. ALRI = cough or difficult breathing and any of: fast breathing, lower chest wall indrawing. CI = confidence interval

**Table 4 Effect of the intervention(s) on mortality, showing the primary analysis (full oxygen period versus pulse oximetry period) and extended analysis (comparing pulse oximetry and full oxygen system periods to the pre-intervention period). Restricted to patients under the age of 5 years (excluding neonates).**

Abstract:

No substantive comments.

Main report:

Introduction:

Well written. No comments.

Methods

Page 5, participants section, lines 129-130. Please clarify what is meant by AGF and AIA enrolled hospitals. Not clear.

**This is referring to particular authors. Reporting guidelines suggest identifying the particular persons responsible for enrolling and consenting participants.**

Page 5, participants section, lines 133-135. How was the 2 week wash in period determined? A priori? Based on certain indicators? Please clarify.

**This was a practical decision based on the feasibility of setting up 3 hospitals in the specified time period. In retrospect, it was not enough time for the pulse oximetry practices to have taken hold, but we did not have any prior data to base this decision on.**

Page 6, Intervention section, lines 153-154. How are hospitals consented? Please clarify.

**Hospitals were invited to send representatives to a major stakeholder meeting, during which the project was presented, discussed. Formal consent for involvement was obtained from representatives following this meeting. Edited text to provide clarity.**

Page 6, Procedures section. How much support was provided during the pulse oximetry period? How much training, supervision etc.? Was this comparable to the full oxygen system support? I'm assuming that training regarding the use of pulse oximeters and target population for measurement were included in the distribution of the devices?

**The training is described in more detail in the Appendix. The basic pulse oximetry training was brief (~1 hour), provided to a few staff who were on duty and available, and focused on the technical use of pulse oximeters and provision of an oximetry/oxygen flow chart (stepping nurses through how to use the oximeter and how to interpret results). The full training was longer (half a day), delivered over multiple sessions to large numbers of staff, and involved more detailed problem-based learning on oximetry, clinical use of oxygen, and oxygen concentrators, including scenarios. The project team visited most hospitals within 2-3 months of distributing the oximeters to trouble-shoot problems and remind staff to use the oximeters. The project team kept regular contact with the data collection nurse located at each site.**

Page 7, outcomes, lines 177 to 180. Given oxygen would be most beneficial (presumably) to children who are hypoxemic, why did the authors not choose hypoxemic children and neonates as their source population given I would assume this is the main target population implementation of oxygen systems would aim to benefit? The main analysis was to compare pulse ox to the full oxygen system such that the oxygen saturation levels should be recorded and known for children during these two primary time periods. Please see more detailed comment above.

**See answer to detailed comment above.**

Results

Page 9, lines 260-262. Could the presentation of more severe illness during the pulse oximetry and full oxygen periods be attributed to data quality differences between the prospective and retrospective periods, especially since support was provided during the prospective data collection period to some degree and this may have influenced the practice of recording of clinical signs (hospitals were not masked)? Please include any audit data supporting the notion that the pre-intervention period data is valid.

**All data was extracted from case records after the patients were discharged. Apart from advising healthcare workers to document SpO2 we gave no other instructions about clinical documentation. We conducted data integrity checking to ensure that data collection nurses were accurately extracting data from the case notes. However, we did not attempt to assess whether documentation practices changed over time, and we are limited in what analysis we can do post hoc (as we have no gold standard to compare to).**

**We do have clear information on missing data over the periods, and we have added this to the Results:**

**“We had complete clinical outcome data for 24778/24859 (99.67%) of participants in the pulse oximetry and full oxygen system periods (prospective data collection), and 23812/24117 (98.74%) of participants in the pre-intervention period (retrospective data collection). We had complete practice outcome data for 24534/24859 (98.69%) of participants in the pulse oximetry and full oxygen system periods, and 20677/24117 (85.74%) of participants in the pre-intervention period.”**

**Missing case notes were more of a problem for children (16.8%) than neonates (9.9%) during the pre-intervention period. If case notes were missing, then we could not record the clinical signs on admission, and these participants were excluded from the proportion reported in Table 2. Comparison of outcomes between participants with case notes *present* versus *missing* suggests that those who died were more likely to have missing case notes (see table below). It is therefore likely that those who died would also have been more likely to have signs of severe illness on admission, so it is possible that the pre-intervention proportions of participants with clinical signs (in Table 2) under-estimate the true proportion.**

**However, misclassification of signs of severity would not have influenced the primary analysis (as signs of severity were not included in the model). The severity-adjusted analysis may have been influenced, as the model excluded those participants with missing case notes (as it was impossible to ascertain their severity signs). This may have slightly underestimated the pre-intervention mortality rates (those with missing case notes were more likely to die) and may have biased the effect estimates towards harm/ (or less benefit).**

|  | **Proportion died:**  **case notes available** | **Proportion died:**  **case notes missing** |
| --- | --- | --- |
| **Pre-intervention** | **Neonates = 8.7%**  **Children = 3.7%** | **Neonates = 25.4%**  **Children = 6.3%** |
| **Pulse oximetry period** | **Neonates = 7.4%**  **Children = 3.2%** | **Neonates = 15.6%**  **Children = 14.4%** |
| **Full O2 system period** | **Neonates = 11.3%**  **Children = 4.6%** | **Neonates = 16.7%**  **Children = 45%** |

Page 10, fidelity and variation from the protocol section, lines 268-269. Given there were power supply issues during the full oxygen implementation period, was any data collected recording periods of power interruptions (frequency and length) and outcomes during these interruptions. Could such interruptions be accounted for in the analyses?

**We recorded data on power outages in our project log book. Overall, the alternative power supply provisions for hospitals with faulty/delayed solar power was very effective. Concentrator power outages were generally <5 minutes (while they switched to the backup supply), with a single occasion at one hospital of 30 minutes (while getting the backup generator started). There were no fatalities during any of these episodes. We did not attempt to adjust for this in analysis. So, while this was not as perfect as we had hoped, it did provide continuous access to oxygen therapy throughout the study.**

Page 11, same section, lines 278-279. Please clarify what the costs were for oxygen, whether this varied by hospital, and whether this could/should be accounted for in the analyses? (or maybe it was through the random effects)

**We did not include analysis of, or adjustment for, oxygen-related costs in this study. We did evaluate costs at baseline, as reported in our needs assessment. See table copied below. We checked on oxygen costs periodically during site visits during project implementation, but we did not systematically evaluate it. We will evaluate this again in medium-term follow-up.**

**Costs of oxygen to facilities and patients**

| Item | Median cost (range) Naira | Median cost (range) USD |
| --- | --- | --- |
|  |  |  |
| OXGYEN COSTS TO FACILITY |  |  |
| Refill H-type oxygen cylinder | ~~N~~10,000 (7,510-11,000) | $50.25 (37.74-55.28) |
| Annual cylinder refill cost | ~~N~~756,000 (96,850-2,280,000) | $3,799 (484-11,400) |
|  |  |  |
| COSTS TO PATIENT |  |  |
| 3-day admission for pneumonia   - Admission/Bed Fee - Medication/Supplies - Oxygen (2 days) | **~~N~~21,925 (0-60,500)**  ~~N~~825 (0-7,500)  ~~N~~11,400 (0-21,980)  ~~N~~7,800 (0-48,000) | **$110.18 (0-304.02)**  $4.12 (0-37.70)  $57.29 (0-110.45)  $39.20 (0-241.21) |

Note: June 31 2015 exchange rate $1 USD: ~~N~~199 Naira.

Page 11, lines 281-283. How did the analysis account for patients who left against medical advice? Presumably this may mean that hypoxemic children at high risk of death left the hospital early and died at home, and these outcomes could have been missed?

**We recorded five possible outcomes:**

- **Discharged well**
- **Died in hospital**
- **Discharged unwell, expected to die**
- **Discharged against medical advice (DAMA) / Abscond**
- **Transfer**

**For the purpose of analysis, death was defined as “died in hospital OR discharged unwell, expected to die”. We observed moderately high rates of discharge against medical advice, driven by user-fees. The proportion of participants that discharged against medical advice did not change significantly between study periods. We did not do any post-discharge follow up to ascertain outcomes.**

**We did collect data on the last SpO_2_ documented prior to discharge/death. This suggests that those that discharged against medical advice may have been sicker than those who were discharged well, but they were not at sick as those transferred out or died.**

- **Among participants that discharged well, 1% had last recorded SpO_2_ <90%.**
- **Among participants who died, 62% had last recorded SpO_2_ <90%.**
- **Among participants that discharged against medical advice or absconded, 9% had last recorded SpO_2_ <90%.**
- **Among participants that were transferred out, 26% had last recorded SpO_2_ <90%.**

**For the reviewer’s interest, we have re-analysed excluding those that discharged against medical advice or absconded.**

|  |  | **Mixed-model adjusted odds ratio (95% CI)** α | | | | | | | |  |
| --- | --- | --- | --- | --- | --- | --- | --- | --- | --- | --- |
|  | **Deaths** | **Primary analysisǂ** | | | | **Extended analysis§** | | | | **ICC**β |
|  | **n/N (%)*** | **Basic model** | | **severity-adjusted** | | **Basic model** | | **severity-adjusted** | | **(95%CI)** |
| **Exclude DAMA/Abscond** | |  | **P** |  | **P** |  | **P** |  | **P** |  |
| Child   - Pre-intervention - Pulse oximetry - Full O_2_ system | 632/13974 (4.5)  227/5986 (3.8)  422/8562) | -  1.07 0.77-1.47) | 0.690 | -  1.16 (0.82-1.66) | 0.402 | -  1.05 (0.69-1.62)  1.01 (0.74-1.38) | 0.808  0.933 | -  1.39 (0.75-2.57)  1.07 (0.68-1.69) | 0.299  0.759 | 0.05  (0.02-0.11) |
| Child ALRI   - Pre-intervention - Pulse oximetry - Full O_2_ system | 116/1751 (6.6)  44/710 (6.2)  97/1096 (8.9) | -  1.10 (0.50-2.44) | 0.814 | -  1.39 (0.59-3.23) | 0.449 | -  0.42 (0.16-1.09)  0.53 (0.29-0.97) | 0.073  0.039 | -  0.29 (0.10-0.83)  0.47 (0.24-0.92) | 0.021  0.028 | 0.10  (0.04-0.23) |
| Neonate   - Pre-intervention - Pulse oximetry - Full O_2_ system | 913/7523 (12.1~~)~~  204/2197 (9.3)  486/3637 (13.4) | -  1.42 (1.02-1.98) | 0.037 | -  1.71 (1.18-2.48) | 0.005 | -  0.89 (0.59-1.33)  0.98 (0.74-1.29) | 0.560  0.891 | -  0.95 (0.59-1.52)  0.94 (0.68-1.31) | 0.828  0.723 | 0.14  (0.05-0.33) |
| Preterm/LBW neonate   - Pre-intervention - Pulse oximetry - Full O_2_ system | 326/1602 (20.4)  82/524 (23.4)  203/867 (23.4) | -  1.46 (0.84-2.53) | 0.175 | -  2.00 (1.10-3.63) | 0.022 | -  1.38 (0.71-2.67)  1.14 (0.73-1.76) | 0.342  0.565 | -  1.11 (0.53-2.30)  1.09 (0.67-1.78) | 0.779  0.732 | 0.10  (0.04-0.25) |

Intervention effects, primary outcomes, page 10. Given the study was so under-powered it is not surprising that the study failed to reject the null hypothesis and its findings were inconclusive. Please add the severity adjusted analyses OR and aOR to the results text here even though there was no qualitative difference to the basic model.

**Revised as requested.**

Given the initial study design intended to compare pulse oximetry to the full oxygen system, why is the primary outcome of death among all children, children with ALRI, and neonates and preterm regardless of their oxygen saturation? Shouldn't the primary target population be children who are hypoxemic? Please see more detailed comment above.

**See previous answer regarding the Hypoxaemic subgroup.**

Page 11, practice outcomes, lines 304-305. Please explain or expand on this sentence indicating that pulse oximetry practices improved by oxygen practices did not.

Page 11, practice outcomes, lines 306-307. Same comment as above. Please clarify this sentence. How specifically did practices improve or not improve.

**Thank you. We have revised this section thoroughly:**

***Practice outcomes***

**Table 5 shows the crude rates and intervention effect sizes for the practice outcomes, including the extended analysis across all three study periods. Pulse oximetry practices improved dramatically across the study periods (from <5% of admissions to >90%), with lesser change evident for oxygen practices.**

**For neonates, results suggest that the full oxygen system may have increased overall oxygen use (aOR 1.34; 0.96-1.87; p=0.085) but did not change oxygen administration for neonates with hypoxaemia (aOR 0.77; 0.34-1.75; p=0.531) compared to the pulse oximetry period. Pulse oximetry had minimal impact on overall oxygen use (aOR 1.29; 0.82-2.03; p=0.264) or the use of oxygen for neonates with signs of hypoxaemia (aOR 1.06; 0.54-20.9; p=0.857) compared to the pre-intervention period.**

**For children, results suggest that the full oxygen system increased overall oxygen use (aOR 1.89; 1.34-2.66; p<0.001) and improved oxygen administration for children with hypoxaemia (aOR 2.71; 1.12-6.56; p=0.027) compared to the pulse oximetry period. Pulse oximetry had minimal impact on overall oxygen use (aOR 0.72; 0.43-1.19; p=0.200) or the use of oxygen for children with signs of hypoxaemia (aOR 1.00; 0.51-1.95; p=0.995).**

**Heat-map graphs suggest that pulse oximetry and oxygen practice changes were achieved incrementally over a number of months, with most of this change occurring early in the pulse oximetry period (Figure 2). Of note, the proportion of hypoxaemic (SpO_2_<90%) children receiving oxygen therapy, increased to >75% coverage within 4-8 months of receiving pulse oximeters. Three hospitals are outliers (H9, H12, and H5’s children’s ward), being the only hospitals that genuinely had no oxygen access for children prior to the intervention and showed no improvement in oxygen use until after the full oxygen system was implemented.**

**Heat-map graphs suggest oxygen use was better targeted at neonates and children with hypoxaemia in the pulse oximetry period, but this is more weakly reflected in the adjusted effect estimates (aOR 2.00; 0.80-4.95; p=0.136 and aOR 1.21; 0.55-2.68; p=0.634 for neonates and children respectively). The ‘indication for oxygen’ outcome uses a composite measure of hypoxaemia (SpO_2_<90% on admission, or signs of hypoxaemia if no SpO2 documented) which may be biased due to the dramatic change in SpO_2_ documentation over the study periods (i.e. reflecting patients with signs of hypoxaemia in the pre-intervention period, and patients with SpO_2_<90% in the latter periods).**

Discussion

Lines 347 to 352, page 12. My main concern with drawing these conclusions is the likelihood of misclassification and also that the practice outcomes seem to be counter to these conclusions. Given the study relied on medical chart extraction and assumed that if a clinical sign was not documented that it was not present, there is likely to be variability in documentation of these signs across the study time period. I would assume that the pre-study period such documentation would be even more variable. I think the authors need to somehow address the potential for misclassification as an important bias in their results, especially the analyses that are using pre-study data that was retrospectively collected. It is further difficult to understand how pulse oximetry would improve outcomes alone when the authors are also reporting data that oxygen use for hypoxemic children did not improve during the pulse oximetry period for both neonates and children. Having a hard time putting all of these pieces together.

Did the authors do any validation exercises of the clinical medical chart to see how reliable the data actually was? This would have been helpful to understand data quality and likelihood of misclassification.

Lines 357 to 360 (and the text following this) in the discussion seem to be directly counter to lines 307 to 309 and lines 311 to 313 in the results section. Please clarify the differences.

**MISCLASSIFICATION. We think the risk of clinical outcome misclassification is low, and we had very little missing clinical outcome data. There is risk of misclassification of clinical signs, and variation between hospitals and over time, but this should not have introduced systematic error (see comments on clinical signs misclassification earlier). Regarding diagnostic classification, a paediatrician assessed the quality of documentation at baseline by reviewing randomly selected case files. We believed the level of detail of clinical signs was adequate to meet diagnostic classifications for ALRI and preterm/LBW. However, we did not conduct clinical observation of doctor’s assessment practices. We preferred to use case definition rather than recorded admission diagnosis for greater objectivity, and because pneumonia is often under-recognised.**

**PRACTICE CHANGE. Heatmap data shows that most hospitals increased their oxygen coverage to >80% of genuinely hypoxaemic children within 4-8 months of pulse oximetry introduction (from <50%) – without substantial changes in overall oxygen use. This change in practice is reflected in the adjusted effect estimates to a lesser extent, within the limitations of practice definitions and analysis models (the mixed-effects model does adjust for variable intervention effect within a study period). See earlier explanation for additional detail.**

Lines 383+ "clinical impact for neonates" section. Very interesting findings. Important.

**Thank you. We have added an additional point to the practical implications to put this in context.**

**“Oxygen, administered as low-flow oxygen or with continuous positive airway pressure (CPAP), is an important part of neonatal care. We advise readers against making changes to neonatal oxygen policies or practices on the basis of this study, and to adhere to WHO guidelines[32]. Oxygen, like any medication, should be given judiciously, accompanied by treatment of the specific medical condition and good supportive care. Future research and quality improvement activities should seek to better understand how oxygen can be used most safely and effectively neonatal care – particularly for the most at-risk preterm neonates.”**
