# Supplementary material for: Oxygen systems to improve clinical care and outcomes for children and neonates: A stepped-wedge cluster-randomised trial in Nigeria
Source: PLoS Med. 2019 Nov 11;16(11):e1002951. doi: 10.1371/journal.pmed.1002951 (PMC6844455; doi:10.1371/journal.pmed.1002951)
Supplement: Attachment — Submitted filename: AuthorReply20191009.docx [file pmed.1002951.s010.docx]

CC: [plosmedicine@plos.org](mailto:plosmedicine@plos.org)

Dear Dr. Graham,

Thank you for resubmitting your manuscript entitled "Oxygen systems to improve clinical care and outcomes for children and neonates: a stepped-wedge cluster-randomised trial in Nigeria." to PLOS Medicine.

At this stage, there are a few remaining issues to address in order to ensure a smooth handover to our production team, should the manuscript be formally accepted.

Can you please address the following issues as soon as possible? Your paper has been sent back to enable you to make these changes.

Article Body

-	Please remove funding, data, protocol, etc from the page after the author summary. The data sharing and funding information should only be in the additional information section of the submission form, not within your manuscript file. Please therefore take the opportunity to ensure those fields are updated in full. Your ethics statement should be in the methods section. The protocol should be in your references list where appropriate.

**Removed as requested.**

-	Please remove the tables/figures list from the end of your manuscript. Only a list of supporting information legends is required.

**Removed as requested.**

-	Please include the corresponding author's email address on the title page of your manuscript, indicated by an asterisk (*). ONLY an asterisk and the email address itself are required. No other author email addresses should be included.

**Removed as requested.**

Figures

-	Please ensure that all main figures are referenced in the manuscript as Fig 1, Fig 2, etc. (including capitalization), rather than Figure 1, Figure 2, etc. Please note, however, that the file names themselves must not include the space, i.e. Fig1.tif, Fig2.tif.

**All main figures are referenced as Fig 1, Fig 2. Files updated as Fig1 and Fig2.**

Tables/boxes

-	Table 1 is formatted as a Box. Please change to “Box 1” and rename other tables accordingly. Please update any in-text citations of the table to Box 1 as well.

**Updated as requested.**

-	Please ensure table titles and legends are outside of the tables themselves. For more information on Tables please see <https://journals.plos.org/plosmedicine/s/tables>

**All table titles and legends are outside the tables.**

Metadata

-	You have added Olatayo Olantinwo as an author since the last revision. PLOS follows the Committee on Publication Ethics (COPE: <https://publicationethics.org/>) guidelines for the addition/removal of authors. We therefore require that any changes to the author listing on a manuscript are confirmed and agreed by all authors, including the authors being removed or added. Please could you contact all authors - including the authors being removed/added - to obtain their emailed confirmation that they agree to this change, and then forward all these confirmations to us in one PDF file?

Please note that all authors must fulfill the ICMJE criteria for authorship. See <http://journals.plos.org/plosmedicine/s/authorship> for details. Please note that we absolutely cannot proceed with your manuscript until we have these agreements.

**We did not add any authors since the last revision. Mr Olatayo was included as an author since the first submission, however there was a typographical error in his name when submitted online which has since been corrected.**

-	Please provide a detailed Financial Disclosure statement on the submission form in full sentences, and include specific grant numbers wherever possible. Currently, the financial disclosure does not begin in full sentences (ie. “This study was funded by…”

**Updated to be in full sentences.**
